# Validating expression of beta cell maturation-associated genes in human pancreas development

**DOI:** 10.3389/fcell.2023.1103719

**Published:** 2023-02-01

**Authors:** Daniel M. Tremmel, Anna E. Mikat, Sakar Gupta, Samantha A. Mitchell, Andrew M. Curran, Jenna A. Menadue, Jon S. Odorico, Sara Dutton Sackett

**Affiliations:** University of Wisconsin-Madison, Department of Surgery, Transplantation Division, Madison, WI, United States

**Keywords:** islets, maturation, development, human, stem cells

## Abstract

The identification of genes associated with human pancreatic beta cell maturation could stimulate a better understanding of normal human islet development and function, be informative for improving stem cell-derived islet (SC-islet) differentiation, and facilitate the sorting of more mature beta cells from a pool of differentiated cells. While several candidate factors to mark beta cell maturation have been identified, much of the data supporting these markers come from animal models or differentiated SC-islets. One such marker is Urocortin-3 (UCN3). In this study, we provide evidence that *UCN3* is expressed in human fetal islets well before the acquisition of functional maturation. When SC-islets expressing significant levels of *UCN3* were generated, the cells did not exhibit glucose-stimulated insulin secretion, indicating that *UCN3* expression is not correlated with functional maturation in these cells. We utilized our tissue bank and SC-islet resources to test an array of other candidate maturation-associated genes, and identified CHGB, G6PC2, FAM159B, GLUT1, IAPP and ENTPD3 as markers with expression patterns that correlate developmentally with the onset of functional maturation in human beta cells. We also find that human beta cell expression of ERO1LB, HDAC9, KLF9, and ZNT8 does not change between fetal and adult stages.

## Graphical Abstract

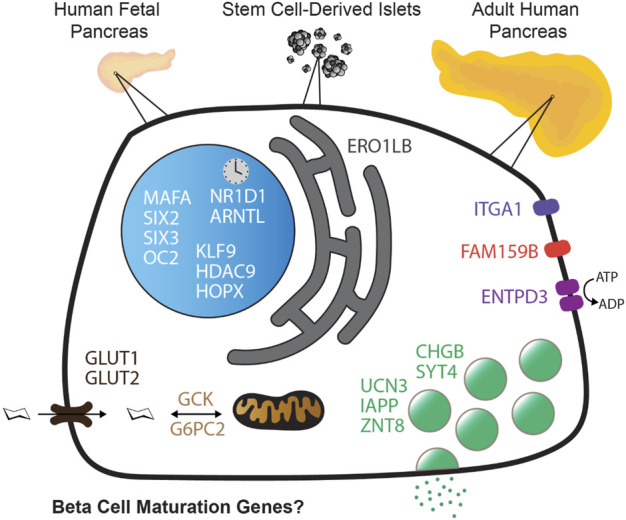

## Introduction

The pancreas is an essential organ composed of exocrine and endocrine cell types. The clusters of endocrine cells, called islets of Langerhans, contain alpha, beta and delta cells, which control blood glucose through the secretion of glucagon, insulin and somatostatin, respectively. Loss of beta cell mass causes diabetes, either due to autoimmune infiltration (Type 1 Diabetes, T1D) or due to stress and damage to the beta cells (Type 2 Diabetes, T2D). Due to a near-inability for mature human beta cells to proliferate (Wang et al., 2015), beta cell loss is irreversible and is treated clinically with exogenous insulin treatment, or beta cell replacement, including either pancreas or islet transplantation. Exogenous insulin administration is an effective treatment yet is imperfect because, unlike endogenous beta cells, it is unable to continuously monitor and prevent hyper- and hypoglycemic episodes. Pancreas and islet transplantation provide a sustainable allogeneic replacement for the patient’s lost or dysfunctional beta cells and can achieve endogenous autoregulation of glycemia. However, a shortage of donor organs, invasiveness of the surgery, inadequate long-term function, and the need for prolonged immunosuppression therapy have made transplantation unavailable to the majority of the afflicted population (Salinno et al., 2019).

As an alternative to transplantation from deceased organ donors, many research groups are focusing efforts towards using human pluripotent stem cells (hPSC), which have the potential to differentiate into insulin-secreting beta cells. *In vitro* differentiation protocols produce “beta-like” cells that express many beta cell genes and can secrete insulin in response to glucose, but based on a combination of analyses including gene expression, metabolic profiles, total insulin content and insulin secretion in response to a variety of stimuli, these cells do not fully resemble primary mature beta cells (Tremmel et al., 2019). Stem cell-derived islet-like clusters (SC-islets) have been shown to further mature following transplantation into mice (Nair et al., 2019; Augsornworawat et al., 2020; Balboa et al., 2022). However, there are also populations of off-target cells (e.g. ductal, acinar, enterochromaffin-like, mesenchymal, and other non-endocrine cells) present in SC-islets (Schulz et al., 2012; Veres et al., 2019; Augsornworawat et al., 2020) which are undesirable to co-transplant with the endocrine cells due to added variables and uncertainty as to how those cells may affect transplant outcomes. In recent clinical trials conducted by ViaCyte in which stem cell-derived pancreatic progenitor cells were transplanted into diabetic patients, the cells secreted stimulated C-peptide and lowered blood glucose after 26 weeks *in vivo*. However, the transplanted cells also differentiated into a variety of off-target cell types and did not have a proper ratio of alpha to beta cells (Ramzy et al., 2021; Shapiro et al., 2021). For application in a clinical setting, SC-islets ideally should have rapid and full function, similar to primary islets. Mature function is necessary for adequate regulation of blood sugar homeostasis; unregulated over-secretion of insulin can lead to hypoglycemia while under-secretion by immature cells during levels of high blood glucose can result in sustained hyperglycemia. To date, no published differentiation protocol has generated fully functionally mature beta cells *in vitro* with levels of total insulin content, glucose-stimulated insulin secretion (GSIS), and gene expression profiles comparable to adult human islets (Tremmel et al., 2019), but the field is continuously moving closer to attaining this goal (Balboa et al., 2022; Barsby and Otonkoski, 2022). The goal of achieving consistent functional maturation of SC-islets *in vitro* and prior to transplantation is a high priority, and would fully realize their potential in clinical and drug discovery applications. To this end, identifying reliable phenotypic markers of beta cell maturity will help to facilitate the characterization of the most effective differentiation protocols.

Compared to adult islets, fetal islets are less responsive to high glucose concentrations, but do respond to other signals including amino acids (Helman et al., 2020). Functional maturation occurs postnatally, which involves the acquisition and gradual enhancement of GSIS until puberty (Otonkoski et al., 1988; Arda et al., 2016). Additional metabolic, epigenetic, and gene expression differences from immature to mature adult beta cells can be identified with cell markers, however they are still not fully understood (Aye et al., 2010; Barsby and Otonkoski, 2022). Stepwise protocols to generate SC-islets use the expression of genes and phenotypic markers at each stage as checkpoints for confirming developmental progress and specification. Understanding these gene expression profiles for normal beta cell development has been key to recapitulating those developmental stages *in vitro*. For example, the discovery that *PDX1* and *NKX6.1* expression must precede *NGN3* induction in order to successfully produce endocrine progenitors has allowed for the generation of more highly enriched endocrine cell and beta cell populations at the end of the differentiation protocol (Nostro et al., 2015). The identification of similar markers at the transition from immature to mature beta cells would provide an efficient way to isolate and purify functionally mature SC-islets from heterogeneous populations, and advance our understanding of the biology underlying maturation.

One example of a gene thought to mark beta cell maturation is Urocortin-3 (UCN3), a peptide hormone that was first identified in mouse pancreatic beta cells in 2003 (Li et al., 2003) and found to be secreted with stimulation by the same secretagogues as insulin (Li et al., 2003; Li et al., 2007). Ucn3 was later identified as a marker of beta cell functional maturation in mice, and found to be expressed only in mouse beta cells postnatally (Blum et al., 2012). In primate and human islets, however, *UCN3* is expressed in both alpha and beta cells (van der Meulen et al., 2012), and has been described as being a marker of human beta cell maturation despite evidence that *UCN3* may be expressed prenatally (Hrvatin et al., 2014; van der Meulen and Huising, 2014). Nevertheless, UCN3 has commonly been used as a tool for monitoring beta cell maturation in SC-islets. None of the leading protocols in the SC-islet field have described significant *UCN3* expression in human stem cell-derived beta or alpha cells (Nair et al., 2019; Velazco-Cruz et al., 2019; Hogrebe et al., 2020; Balboa et al., 2022), although some have observed expression of UCN3 following transplantation of SC-islets and subsequent maturation *in vivo* (van der Meulen and Huising, 2014; Balboa et al., 2022). UCN3 is secreted along with insulin, packaged within the same secretory vesicles (van der Meulen et al., 2015), and is active in a paracrine feedback mechanism in which it binds corticotropin releasing hormone receptor 2 (CRFR2) on delta cells to stimulate somatostatin secretion, which inhibits alpha and beta cell function (van der Meulen et al., 2015). Loss of *UCN3* has also been associated with type 2 diabetes in mice (Blum et al., 2014) and in humans (van der Meulen et al., 2015). *Ucn3* KO mice were recently generated and reported to have no deficit in beta cell maturation or function, deeming Ucn3 a marker, but not a driver of maturation (Huang et al., 2020).

Many other maturation markers have been postulated through studies of mouse development, human diabetes, or by studying human SC-islets, but lack corresponding evaluation comparing mature adult vs. immature fetal human tissues (Zito et al., 2010; Veres et al., 2019; Barsby and Otonkoski, 2022). Arda et al. provided an important resource with their 2016 publication assessing single-cell human beta cell transcriptomes, identifying several genes that significantly change expression during early postnatal development (Arda et al., 2016). Interestingly, this study did not identify *UCN3* as a gene of interest; instead *MAFA*, *SIX2*, *SIX3* and several other genes were identified to have moderate, but significant, increases in expression between juvenile and adult human beta cells. The Arda et al. study has been cited to justify low expression of genes such as *MAFA* in stem cell-derived islets to be similar to neonatal beta cells, but the relative expression profiles are not comparable. For example, whereas *MAFA* expression from juvenile to adult beta cells was found to have approximately a two-fold increase, primary human islets have at least 10-fold higher *MAFA* expression than SC-islets (Nair et al., 2019; Tremmel et al., 2019; Velazco-Cruz et al., 2019). The more recent differentiation protocol described by Balboa et al., with extensive characterization of SC-islet function and metabolism, also found that *MAFA* and *UCN3* are not highly expressed *in vitro* but only increase in expression to levels comparable to primary islets weeks after transplantation (Balboa et al., 2022). Furthermore, it is important to validate these markers by protein expression as well as gene expression, as it is well known that the presence of gene transcripts does not necessarily correlate with protein expression (Vogel and Marcotte, 2012). For example, previous studies have found that alpha and beta cells both express RNA encoding *INS* and *GCG*, although at the protein level INS is only expressed by beta cells, and GCG only by alpha cells (Blodgett et al., 2015).

Here we report two SC-islet differentiation protocols, one which generates alpha and beta cells that express *UCN3* at levels comparable to native adult islets, but still lack function comparable to primary islets. We explore the expression profiles of *UCN3* in SC-islets as well as in primary human fetal pancreas and adult islets to establish that *UCN3* expression does not correlate with functional maturation in human islets. We then explore the expression profiles of other candidate beta cell maturation markers in human fetal and adult tissues to help evaluate the relevance of these genes and proteins in normal human islet development.

## Results

**FIGURE 1:**
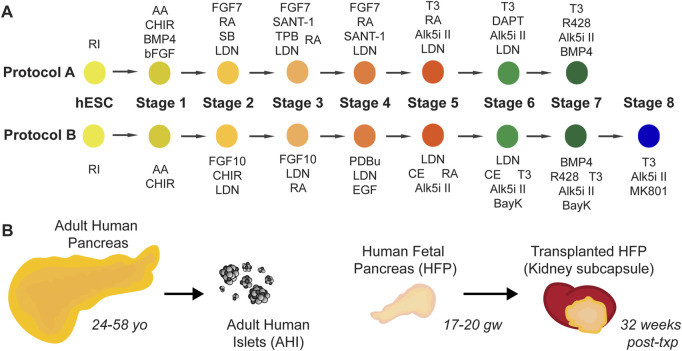
Resources used in this study. **(A)** Schematic diagrams for two SC-islet differentiation protocols, Protocol A (“A”) and Protocol B (“B”). Full details of these protocols are detailed in Supplemental Tables 1-3. **(B)** Adult human pancreas tissue (age range 24–58 years old), isolated adult human islets (“AHI”), human fetal pancreas (“HFP”) (age range 17–20 gestational weeks) and matured HFP grafts (32 weeks post-transplant) are used in this study for assessment of gene expression, protein localization, and function.

Two protocols developed in our lab, at a time before dynamic insulin secretion was demonstrated in SC-islets, are outlined in Figure 1A and detailed fully in Supplemental Tables S1–3. Protocol A was derived from our previously published protocol (Xu et al., 2011), with modifications in the late stages of differentiation based on (Rezania et al., 2014) and (Pagliuca et al., 2014). Protocol B was further modified using Stages 1–4 from Nostro et al. (2015) to derive an improved pancreas progenitor population, and with factors such as BayK 8644 and MK-801 added to the end stages influenced by (Zhu et al., 2016)and (Huang et al., 2017). Although SC-islets from both protocols, Protocol A and Protocol B, have relatively low glucose-responsive function, we find that major gene expression differences between these two protocols warrant the use of these SC-islets for further study. To complement these protocols, our study utilizes banked human tissues to investigate candidate human beta cell maturation markers in human pancreas development: adult human pancreas tissue, isolated adult human islets (AHI), human fetal pancreas (HFP), and HFP grafts transplanted in mice and matured for 32 weeks of additional *in vivo* development (Figure 1B).

### UCN3 expression in SC-islets does not correlate with improved function

**FIGURE 2:**
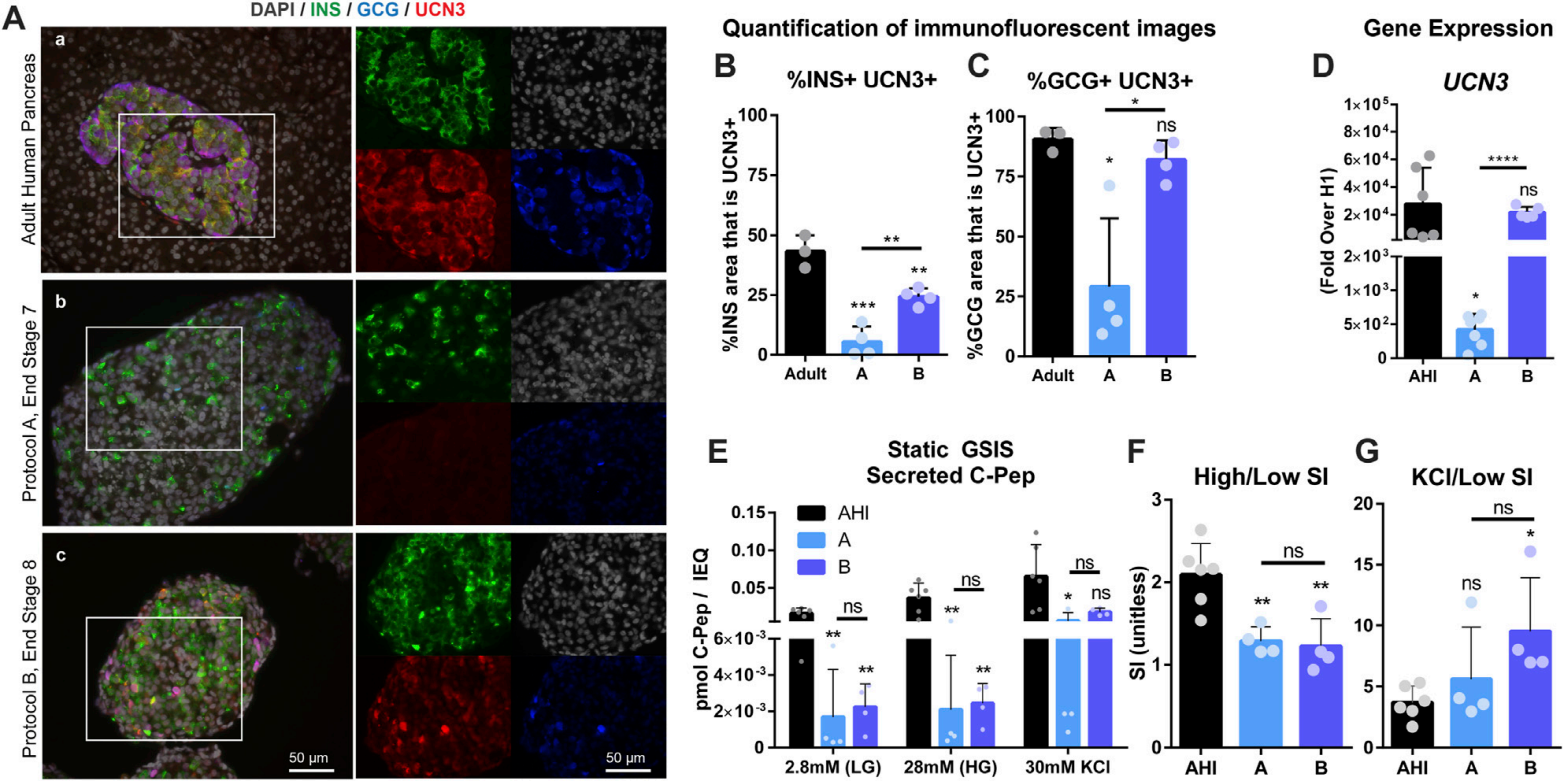
UCN3 expression in SC-islets does not correlate with improved function. **(A)** Immunostaining for insulin (green), glucagon (blue) and UCN3 (red) in adult human pancreas **(A)**, and end-stage cells collected after differentiation using Protocols A **(B)** and B **(C)**. Images of individual channels are the same magnification as the merged images. Scale bars are 50 microns. **(B)** Quantification of co-localization of UCN3 with insulin in adult human pancreas, Pro A or Pro **(B)**. **(C)** Quantification of co-localization of UCN3 with glucagon in adult human pancreas, Pro A or Pro **(B)**. **(D)** Gene expression of *UCN3* normalized to beta-actin, in adult human pancreas and SC-islets derived *via* Pro A or Pro B relative to undifferentiated cells (H1). **(E)** Static GSIS assay to assess insulin secretion in response to low glucose (LG, 2.8 mM), high glucose (HG, 28 mM) and KCl (30 mM) measured by the concentration of human C-Pep in the supernatant (pmol C-Pep/IEQ). **(F)** Glucose-mediated stimulation index (SI) (C-pep secreted under high glucose/C-pep secreted under low glucose) calculated from the static GSIS assay. **(G)** Potassium-mediated SI (C-pep secreted under KCl/C-pep secreted under low glucose) calculated from the static GSIS assay. Statistical indicators directly above each bar are compared to adult pancreas or AHI (ns = not significant, *p < 0.05, **p < 0.01, ***p < 0.001, ****p < 0.0001).

Human embryonic stem cells (hESCs) were differentiated to the end of Protocol A (Pro A, end Stage 7, day 28) and Protocol B (Pro B, end Stage 8, day 28) to assess gene expression, protein localization and SC-islet function. UCN3, insulin (INS), and glucagon (GCG) were co-stained to reveal that Pro A has very low levels of UCN3, while Pro B has higher UCN3 expression in both INS^+^ and GCG^+^ cells, similar to *in situ* adult human islets (Figure 2A). Co-localization of UCN3 with either INS (Figure 2B) or GCG (Figure 2C) was quantified to find that in Pro B, UCN3 is expressed in alpha cells at a similar level as in adult pancreas, while UCN3 is expressed in beta cells at a reduced level compared to adult pancreas, but at a significantly higher level than in Pro A. Relative *UCN3* gene expression in Pro B was insignificantly different from AHI, whereas Pro A had low *UCN3* gene expression which was significantly lower than both Pro B and AHI (Figure 2D). Despite Pro B expressing substantially improved UCN3 levels, the functional performance of Pro A and B in a static GSIS assay were not distinguishable from one another (A, Avg SI = 1.3; B, Avg SI = 1.2), and both performed poorly compared to isolated AHI (Avg SI = 2.2) (Figures 2E,F). Despite having poor insulin secretion in response to glucose, SC-islets were responsive to potassium stimulation (Figure 2G). The gene expression profiles for SC-islets derived from these two differentiation protocols compared to adult human islets is included in Supplemental Figure S1, and additional characterization is included in Supplemental Figure S2.

### UCN3 is highly expressed in human fetal islets, long before attainment of functional maturation

**FIGURE 3:**
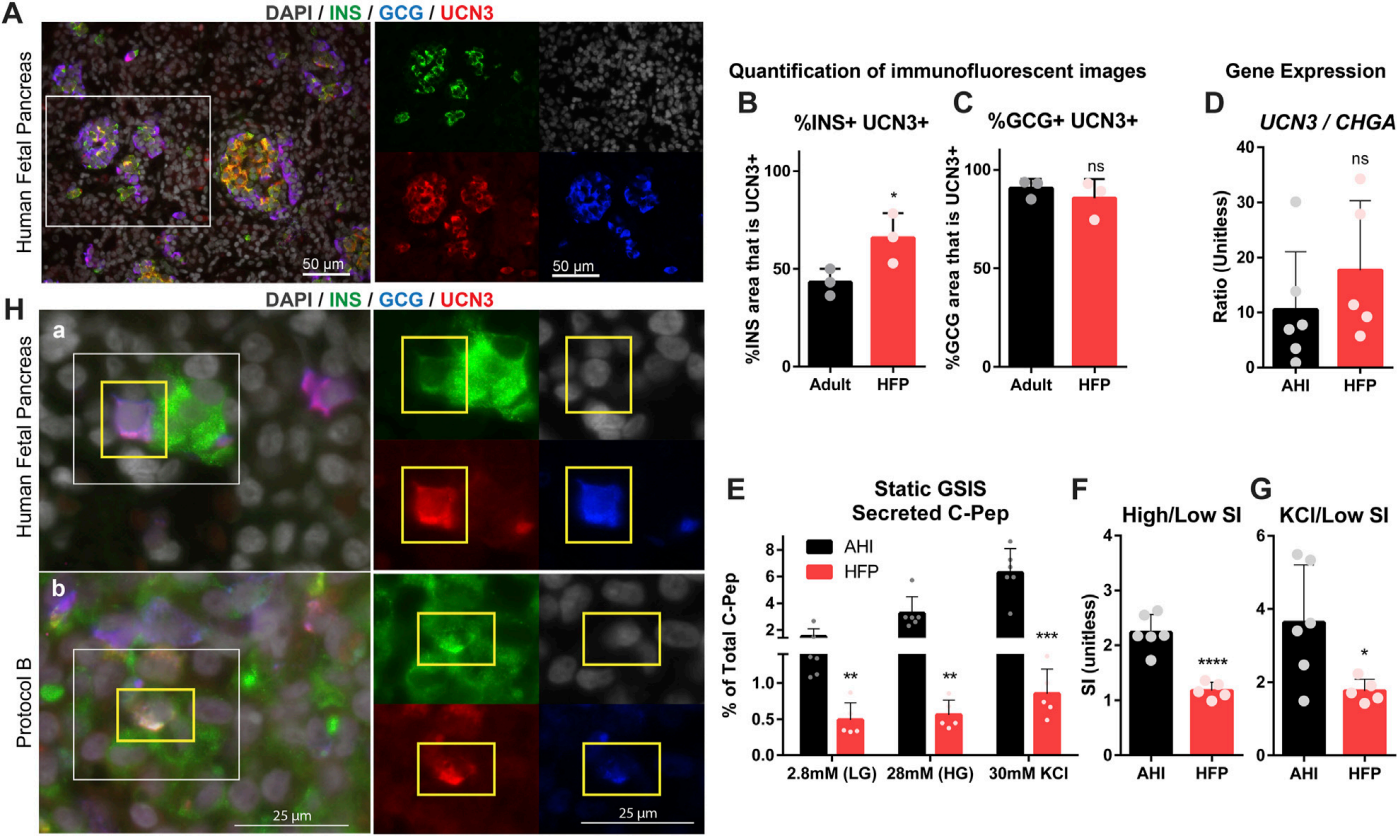
UCN3 is highly expressed in human fetal islets, long before attainment of functional maturation. **(A)** Immunostaining for insulin (green), glucagon (blue) and UCN3 (red) in human fetal pancreas (HFP). Scale bars are 50 microns. **(B)** Quantification of co-localization of UCN3 with insulin in adult human pancreas and HFP. **(C)** Quantification of co-localization of UCN3 with glucagon in adult human pancreas and HFP. **(D)** Gene expression of *UCN3* normalized to *CHGA* in adult human islets (AHI) and HFP. **(E)** Static GSIS assay to assess insulin secretion in response to low glucose (LG, 2.8 mM), high glucose (HG, 28 mM) and KCl (30 mM) measured by the concentration of human C-Pep in the supernatant as a percentage of total C-Pep content. **(F)** Glucose-mediated stimulation index (SI) (C-pep secreted under high glucose/C-pep secreted under low glucose) calculated from the static GSIS assay. **(G)** Potassium-mediated SI (C-pep secreted under KCl/C-pep secreted under low glucose) calculated from the static GSIS assay. To normalize the number of islets loaded into each GSIS replicate and because the exact fetal islet IEQ could not be counted, GSIS results are presented as a percentage of the total insulin content of each sample. **(H) (A)** In normal HFP, polyhormonal (INS^+^GCG^+^) cells are considered an immature population and can be found to express UCN3 at this developmental stage. **(B)** In SC-islets derived with Protocol B, UCN3^+^INS^+^GCG^+^ triple-positive cells can also be found. Images of individual channels are the same magnification as the merged images. Scale bars are 25 microns. Statistical indicators directly above each bar are compared to adult pancreas or AHI (ns = not significant, *p < 0.05, **p < 0.01, ****p < 0.0001).

To further explore the relevance of UCN3 as a maturation marker in human islets, HFP was compared to adult pancreas tissue and AHI to assess gene expression, protein localization and function. Co-staining of HFP tissues with UCN3, INS, and GCG revealed that UCN3 is strongly expressed in both INS^+^ and GCG^+^ cells at early fetal stages (17–20 gestational weeks) (Figure 3A). Quantification of co-localization of UCN3 with INS (Figure 3B) and GCG (Figure 3C) indicates that most GCG^+^ area co-expresses UCN3 at both developmental time points, while a significant increase in INS^+^UCN3^+^ area was found in HFP compared to adult pancreas.

To assess gene expression of *UCN3* in HFP compared to AHI and SC-islets, *UCN3* and Chromogranin A (*CHGA*) gene expression was measured. HFP contains developing mesenchymal, exocrine, duct and endocrine tissue, while purified adult islets contain primarily endocrine cells, therefore the gene expression levels in these samples requires normalization for comparison. *CHGA* was selected for normalization because it is expressed at high levels in both alpha and beta cells (Supplemental Figure S4A), its expression precedes that of hormones in endocrine cell development, and it is not lost with de-differentiation in beta cell failure. For these reasons, we felt that *CHGA* rather than a gene like *INS* was better for normalization, because insulin expression itself is lower in immature cells and is lost in de-differentiated states (Talchai et al., 2012; Moin et al., 2018; Ramond et al., 2018). Thus, the ratio of *UCN3*/*CHGA* was used to normalize to the fraction of RNA derived from the endocrine population only. The ratio of *UCN3*/*CHGA* was not significantly different between AHI and HFP (Figure 3D). Although it has been well established that prenatal human islets lack mature function, we assessed function in HFP compared to AHI using a static GSIS assay. Not surprisingly, HFP had significantly reduced C-Pep secretion at all stages of the GSIS compared to AHI (Figure 3E). HFP had an average stimulation index (SI) of 1.1, as defined by insulin secreted in high (28 mM) glucose over insulin secreted in low (2.8 mM) glucose (Figure 3F), reflecting no stimulation, while C-Pep secretion was stimulated by potassium (Figures 3E,G).

### UCN3 is expressed in polyhormonal cells in HFP and SC-islets

Polyhormonal cells that co-express multiple endocrine hormones, such as insulin and glucagon, are known to be an immature cell type in the developing pancreas (Jennings et al., 2015). Although rare in HFP at gestational week 17 and older (Riedel et al., 2012), we observed the presence of INS^+^GCG^+^ cells that also co-expressed UCN3 (Figures 3A–H). Polyhormonal cells are also generated by some SC-islet differentiation protocols, and are considered to be an undesirable immature cell type (Nostro et al., 2015; Russ et al., 2015). INS^+^ GCG^+^ UCN3^+^ cells were also found in Protocol B (Figures 3B–H). The existence of INS^+^ GCG^+^ UCN3^+^ in both normal human fetal pancreas and SC-islets is therefore a striking example of how UCN3 is present well before beta cell maturation occurs. The percentage of INS^+^GCG^+^ area in SC-islets derived from Protocols A and B and in HFP is included in Supplemental Figure S2E.

### Gene expression of candidate maturation markers in adult human islets and fetal pancreas

**FIGURE 4:**
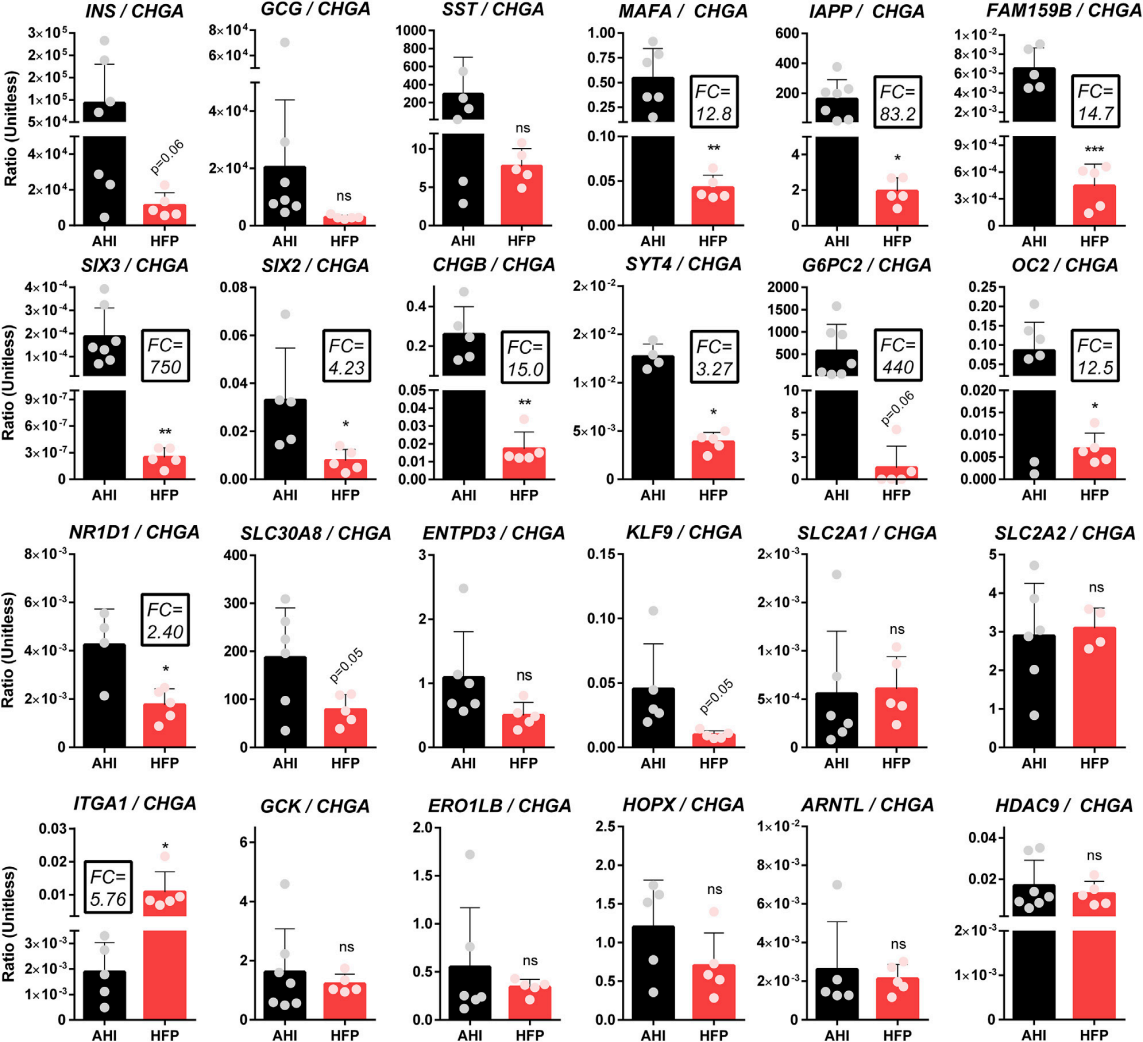
Gene expression of candidate maturation markers in adult human islets and fetal pancreas. QPCR-based analysis of gene expression of several candidate maturation markers in adult human islets (“AHI”) and human fetal pancreas (“HFP”). All genes were normalized to *β* actin within the same sample, then normalized to undifferentiated H1 cells and presented as a ratio of gene expression over *CHGA* to normalize for differences in endocrine mass between the various samples. Fold changes (FC) listed highlight major differences in gene expression between AHI and HFP. Statistical indicators directly above each bar are compared to AHI (ns = not significant, *p < 0.05, **p < 0.01, ***p < 0.001).

Having established the abundant expression of UCN3 prior to islet functional maturation in human tissues, we employed the same resources and methods to interrogate other candidate maturation markers identified in a review of primary islet and SC-islet maturation literature (Hrvatin et al., 2014; Arda et al., 2016; Saunders et al., 2019; Veres et al., 2019; Augsornworawat et al., 2020; Balboa et al., 2022; Barsby and Otonkoski, 2022; Maxwell et al., 2022). Due to the practical applications of quantitative real-time PCR (QPCR) in assessing expression of multiple genes across large numbers of samples, QPCR is often used to evaluate SC-islet differentiations both at the end stage and at distinct stages throughout differentiation. The expression levels of an array of candidate maturation marker genes were measured by QPCR in human fetal pancreas (HFP) (N = 5 donors) and adult human islets (AHI) (N = 5-7 donors); for all genes, *CHGA* was used for normalization to the endocrine population (Figure 4).

The results indicate that several genes (e.g. *ARNTL*, *ERO1LB*, *HOPX*, *SLC30A8*) do not have increased expression from fetal to adult islets, and therefore may not correlate with maturation. In contrast, many genes (e.g. *CHGB*, *FAM159B*, *IAPP*, *MAFA*, *SIX2*, *SIX3*, *SYT4*) do exhibit significant differences in expression between fetal and adult islets that suggests a correlation with maturation. Importantly, this method is only useful for markers that are specific to or highly enriched in islets compared to other pancreas cell types. The fold change (FC) in expression from fetal to adult islets is listed in the figure for genes of particular interest.

### Protein expression and localization during human beta cell maturation

**FIGURE 5:**
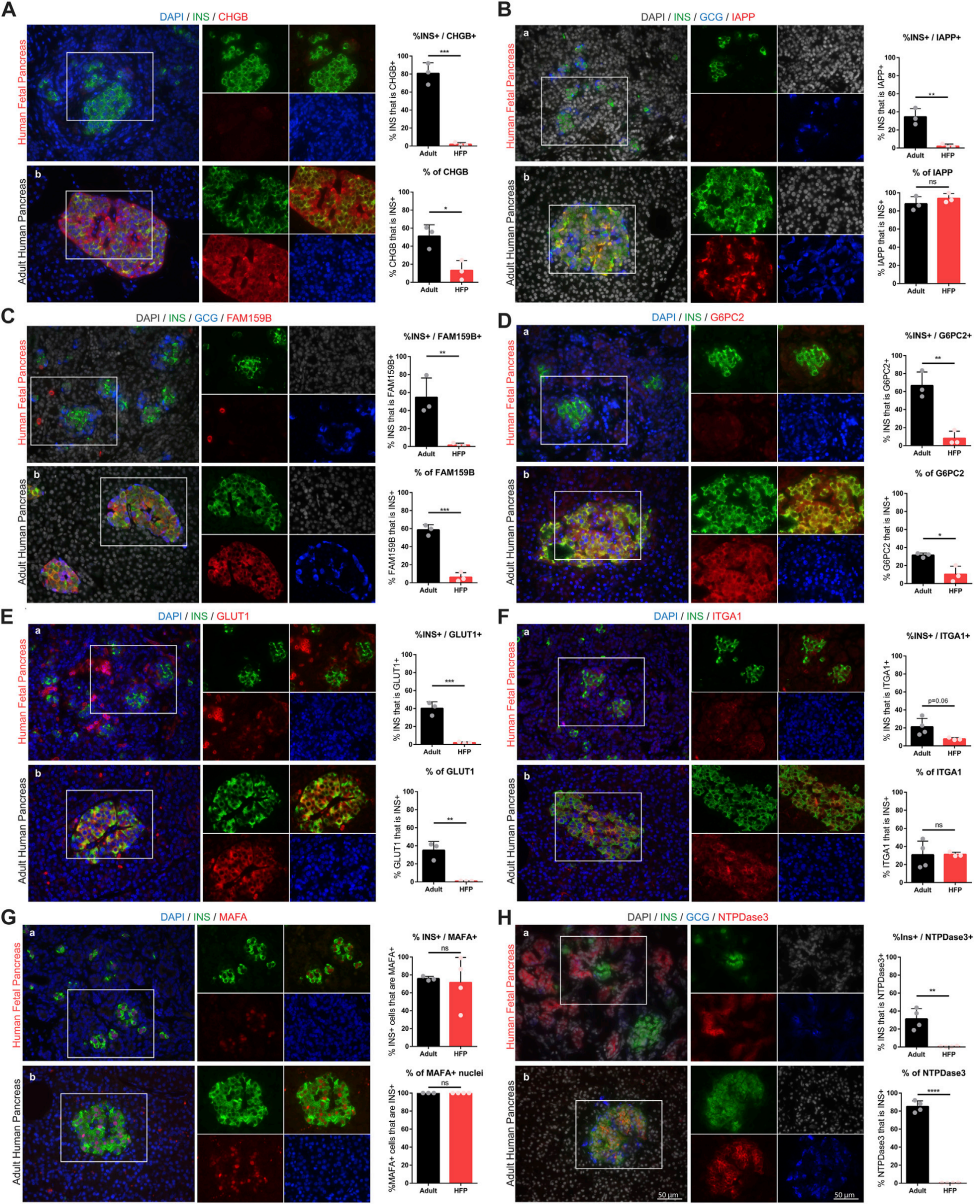
Protein expression and localization during human beta cell maturation. Immunofluorescent staining of candidate maturation markers reveals significant increase in beta cell-specific expression of CHGB **(A)**, IAPP **(B)**, FAM159B **(C)**, G6PC2 **(D)**, GLUT1 **(E)**, ITGA1 **(F)**, MAFA **(G)**, NTPDase3 **(H)** in human fetal and adult pancreas sections. Individual channel images are the same magnification as the larger merged images. Scale bars = 50 microns. For each marker, image quantification is depicted as the percentage of insulin that co-localizes with each marker (top graphs) and the percentage of each marker that co-localizes with insulin (bottom graphs). (ns = not significant, *p < 0.05, **p < 0.01, ***p < 0.001, ****p < 0.0001).

To validate the gene expression data, proteins of interest (POI) were assessed with immunofluorescence (IF) staining on human fetal and adult pancreas sections (Figure 5). Where possible, antibodies used in this study were matched to those of previous studies that established each protein as a marker of maturation. Each candidate marker (red) was co-stained with insulin (green). Images were used to quantify protein localization and expression differences between fetal and adult beta cells, and specificity of the maturation marker expression in islets compared to other pancreatic cell types (such as the exocrine or ductal cells). To assess the expression of each protein in the beta cell population, the Manders coefficient is reported as the percentage of insulin-positive area that is co-positive for the protein of interest (“%INS+/POI+”) (Dunn et al., 2011). To assess the specificity of each marker to the beta cell population, the Manders coefficient is reported as the percentage of area for each marker that is co-positive for insulin (“% of POI”). For markers that localize to the nucleus (e.g. MAFA) these measures were calculated by counting the number of INS + cells with marker + nuclei, and the number of Ins-negative cells with marker + nuclei.

We find that islet amyloid polypeptide (IAPP) is an islet protein with beta cell-specific expression that significantly increases in abundance between fetal and adult stages, but is not expressed in all beta cells. Ectonucleoside triphosphate diphosphohydrolase-3 (NTPDase3, encoded by the gene *ENTPD3*) also has strong beta cell-specific expression in adult islets, and is absent in fetal beta cells, but is expressed in fetal exocrine tissue. We also find that chromogranin B (CHGB), FAM159B, glucose-6-phosphatase catalytic subunit 2 (G6PC2), and glucose transporter 1 (GLUT1) significantly increase expression between the fetal and adult stages, but that these markers are not restricted to only beta cells. Interestingly, we find MAFA is present in most beta cells at both the fetal and adult stages, although it appears to show significantly increased protein expression in adulthood, consistent with increased gene expression (Figure 4). Finally, we find that integrin subunit alpha 1 (ITGA1, also called CD49a), which has been used to enrich a stem cell-derived beta cell population with enhanced function (Veres et al., 2019), has slightly elevated protein expression levels in adult islets but does not appear to be expressed solely by beta cells. Gene expression levels of ITGA1 are lower in isolated adult islets compared to fetal pancreas (Figure 4) and recent single cell RNA sequencing studies have also found that beta cells from isolated human islets have low levels of ITGA1 expression (Balboa et al., 2022). These inconsistent findings may be due to the inherent destruction of ECM during islet isolation (Cross et al., 2017; Meier et al., 2020) which could account for the downregulation in gene and protein expression of integrins, or other ECM-affiliated genes, in cultured islets following isolation. Indeed, using samples banked from various stages of islet isolation, we observe that ITGA1 expression and localization changes during islet isolation and culture compared to islets *in situ* (Supplemental Figure S3).

Additional markers that were assessed and found to have similar protein expression (no significant difference) between fetal and adult beta cells (CHGA, ZNT8, ERO1LB, HDAC9, and KLF9) are included in Supplemental Figure S4.

### Human fetal pancreas functional maturation corresponds with increased maturation marker expression

**FIGURE 6:**
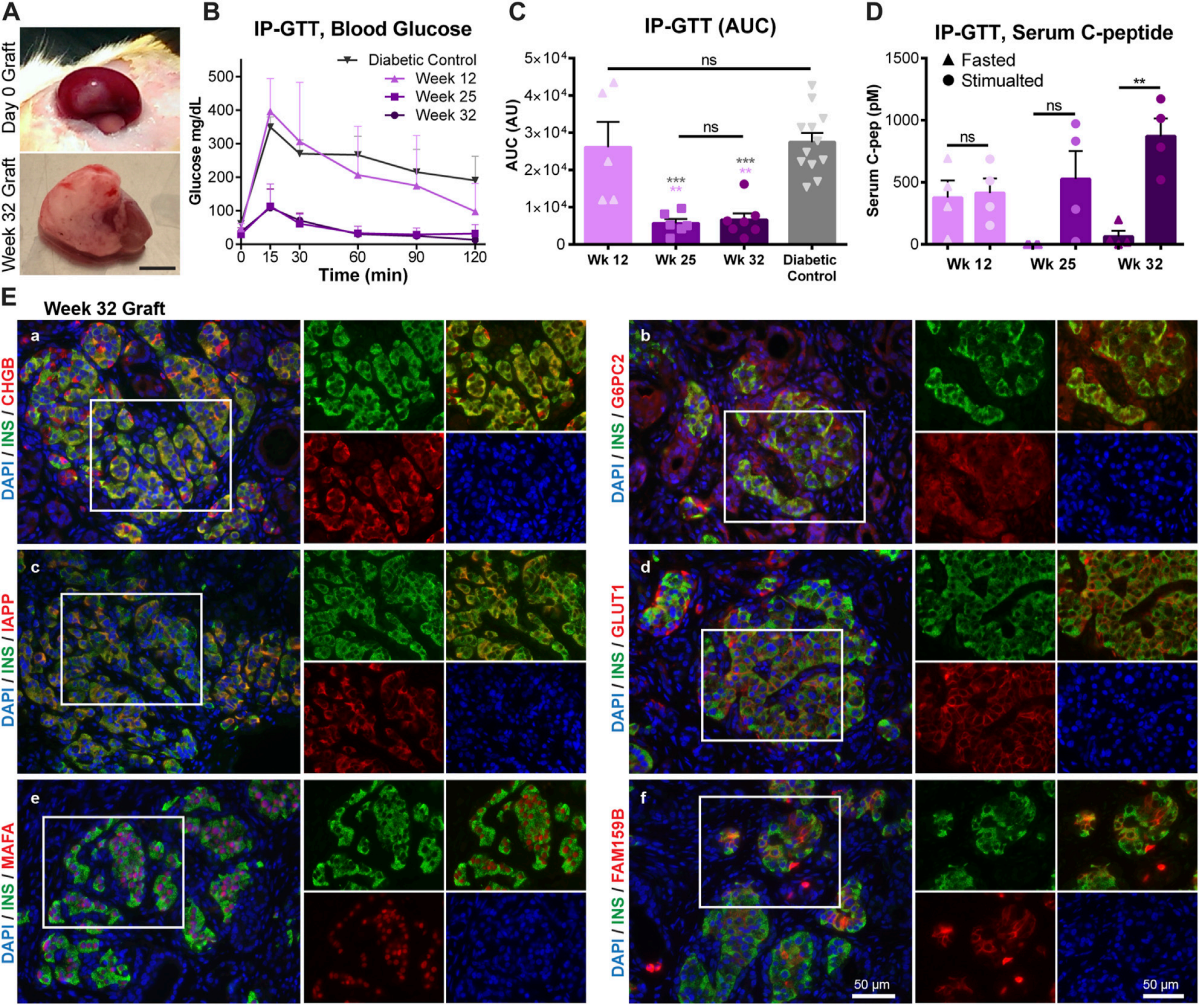
Human fetal pancreas functional maturation corresponds with increased maturation marker expression. Transplanted HFP was monitored for *in vivo* function through IP-GTT. **(A)** Representative images of the graft on the day of transplantation (Day 0 Graft) and after 32 weeks. Scale = 0.5 cm. **(B)** Blood glucose values during IP-GTT at 12, 25, and 32 weeks post-transplantation compared to diabetic non-transplanted controls. **(C)** Area under the curve (AUC) values for the IP-GTT curves. Gray asterisks indicate statistical comparison to the diabetic control, while light purple asterisks indicate statistical comparison to Week 12. **(D)** Serum human C-peptide levels in fasted and stimulated (15 min after glucose injection) states. **(E)** Representative images of immunofluorescent staining of 32 weeks grafts for maturation markers (in red) CHGB (a), G6PC2 (b), IAPP (c), GLUT1 (d), MAFA (e), FAM159B (f). Scale = 50 microns. (ns = not significant, **p < 0.01, ***p < 0.001).

To assess how closely expression of the markers identified in our study correlates with the onset of human islet functional maturation, fresh HFP tissue (17–20 gestational weeks) was transplanted into the kidney subcapsular space (KSC) of diabetic immunodeficient NSG mice and allowed to mature for an additional 32 weeks (Figure 6A). Graft function was assessed at weeks 12, 25 and 32 post-transplantation through an intraperitoneal glucose tolerance test (IP-GTT). After 12 weeks *in vivo*, animals transplanted with HFP were indistinguishable from non-transplanted diabetic control mice in GTT performance and area under the curve (AUC) (Figures 6B,C), but had detectable levels of serum human C-peptide that were not stimulated after glucose injection (Figure 6D), indicative of immature function. At weeks 25 and 32, the GTT performance was significantly improved, with AUC values resembling non-diabetic mice and low fasted blood glucose levels (Figures 6B,C). However, there was a statistically significant difference in the stimulated C-Peptide level after glucose injection at week 32, but not at week 25 (Figure 6D). This indicates that after 32 weeks post-transplantation, the HFP grafts have undergone functional maturation, have controlled insulin secretion in response to glucose and are restoring glucose homeostasis in the transplanted animals.

HFP grafts were recovered after 32–35 weeks post-transplantation for histological analysis. Maturation markers that were found to have low levels of immunostaining in fetal islets, but high protein expression levels in adult islets were assessed in the transplanted grafts. All markers presented (CHGB, FAM159B, G6PC2, GLUT1, IAPP, MAFA) were found to be expressed at high levels, similar to that of the adult beta cells, after the onset of functional maturation *in vivo* (Figure 6E).

## Discussion

The concept of a ‘maturation marker’ in beta cell development may be controversial, as beta cells are a heterogeneous population and many believe one single gene or protein will not be sufficient to define maturation. In this study, we aim to understand how expression profiles of key beta cell genes change in human islets from fetal to adulthood, not to identify one single maturation marker, but to better define the profile of gene and protein expression that accompanies human beta cell maturation. This information is useful not only in understanding normal human beta cell development, but may aid in understanding beta cell function, defining subpopulations of mature human beta cells, identifying changes in expression of these markers with disease, and improving assessment of stem cell-derived islets.

In recent years, significant improvements in SC-islet *in vitro* function have been achieved (Nair et al., 2019; Hogrebe et al., 2020; Balboa et al., 2022). Despite this progress, it is clear that expression of certain genes (e.g. *MAFA, SIX2, SIX3, UCN3*) *in vitro* is deficient compared to primary human islets. After transplantation, the SC-islets undergo change, which includes improved beta cell maturation (Augsornworawat et al., 2020; Balboa et al., 2022; Maxwell et al., 2022), but also involves subpopulations of end-stage cells differentiating into off-target cell types (Augsornworawat et al., 2020). Cell sorting prior to transplantation to purify cells using a variety of different markers has been shown to improve graft outcomes, thereby avoiding the risk of teratoma formation and leading to a decreased population of off-target cell types and a more pure and functional population of pancreatic endocrine cells (Nair et al., 2019; Veres et al., 2019; Aghazadeh et al., 2022; Parent et al., 2022). However, cell sorting is relatively inefficient, requiring a large number of cells and only recovering a fraction of the total targeted population and is stressful and damaging to the recovered cells. The fact that end-stage SC-islets require purification prior to transplantation (Nair et al., 2019; Veres et al., 2019), and the fact that significant biological changes occur in the cells following transplantation (Augsornworawat et al., 2020) suggest that SC-islet differentiation protocols may require further improvements in order to achieve the safe, stable, and functional differentiated *in vitro* product that is desirable for clinical application. A better understanding of phenotypic gene expression profiles that mark a stable, mature beta cell will guide this process.

Our study establishes that UCN3 is not a reliable marker of human beta cell maturation. *UCN3* is highly expressed in fetal human pancreas, in both alpha and beta cells, and can be expressed by immature polyhormonal cells. We also report that high levels of *UCN3* expression in SC-islets does not correlate with an improvement in GSIS function as seen in Protocol B of this study. Together, these data suggest that UCN3 may not be useful to identify a more mature population of SC-islets, but intriguingly also reveals that *UCN3* expression is abnormally delayed in almost every published SC-islet differentiation protocol, where SC-islets resemble fetal-like beta cells in some ways but *UCN3* gene expression remains nearly undetectable (Nair et al., 2019; Hogrebe et al., 2020; Balboa et al., 2022). If UCN3 is expressed in immature alpha and beta cells during normal development, it may have an unknown role in the cells at these stages that has yet to be identified. Our data showing UCN3 gene and protein expression in human fetal islets corroborates previous data sets that have also indicated that *UCN3* is expressed in human fetal islets (Hrvatin et al., 2014; van der Meulen and Huising, 2014; Blodgett et al., 2015); non-etheless *UCN3* has continued to be touted as a maturation marker in many studies (Barsby and Otonkoski, 2022).

V-maf musculoaponeurotic fibrosarcoma oncogene homolog A (MAFA) is a transcription factor critical for beta cell development, which binds to the insulin gene enhancer/promoter region and drives insulin expression in response to glucose (Zhu et al., 2017). We find that *MAFA*, which has also been particularly under-expressed in SC-islets from many different protocols (Nair et al., 2019; Velazco-Cruz et al., 2019; Hogrebe et al., 2020; Balboa et al., 2022), is expressed in early fetal human beta cells, and has only a 12.8-fold increase in gene expression from fetal to adult stages (Figure 4). While previous studies found that *MAFA* expression increases 2-fold during postnatal maturation from human neonatal to adult beta cells (Arda et al., 2016), SC-islet *MAFA* expression in our study was on the scale of 100 to 250-fold lower than primary human islets, and significantly lower than that of human fetal islets (Supplemental Figure S1B, Supplemental Figure S5). This suggests that the extremely low *MAFA* expression at the final stage of differentiation in our protocols, as well as many others in the field (Nair et al., 2019; Velazco-Cruz et al., 2019; Hogrebe et al., 2020; Balboa et al., 2022), may be indicative of incomplete commitment to a beta cell fate. The significant deficit in SC-islet *MAFA* expression cannot be ignored or justified based on the minor increase in beta cell *MAFA* expression between birth and adulthood in human development.

In addition to *MAFA*, the genes *SIX2* and *SIX3* were identified by Arda *et al.* to exhibit significantly increased expression as human beta cells transition from neonatal to adult stages (Arda et al., 2016). In particular, *SIX2* and *SIX3* have also been identified to affect GSIS function in SC-islets and primary human islets (Velazco-Cruz et al., 2020; Bevacqua et al., 2021; Balboa et al., 2022). Balboa et el. found that while *SIX2* expression was present during *in vitro* SC-islet differentiation, *SIX3* was not expressed even weeks following transplantation (Balboa et al., 2022). In our study, we find that the relative gene expression of *SIX2* and *SIX3* are significantly lower in fetal compared to adult pancreas, which supports the notion that they represent potential maturity markers. Unfortunately, due to discontinuation of commercially available antibodies for SIX3, we were unable to assess protein expression of this marker. We found that a commercially available antibody against SIX2 previously used in other studies has high levels of non-specific binding in the pancreas. However, quantification of nuclear localization of SIX2 using this antibody suggests that *SIX2* expression is significantly increased in adult compared to fetal beta cells, and correlates with the functional maturation of transplanted HFP (Supplemental Figure S6). Better antibodies for SIX2 and SIX3 will need to be developed to further study these proteins *in situ* in human tissue sections.

Our data corroborates previous findings that NTPDase3 (encoded by the gene *ENTPD3*) is a beta cell maturation marker in human development (Figure 5H) (Saunders et al., 2019). *ENTPD3* has also been shown to mark a functionally mature subset of SC-islets and is useful for selecting that subset from the total population of differentiated cells (Docherty et al., 2021). Changes in glucose-sensitive purine synthesis and metabolic ATP trafficking that occur with beta cell maturation in postnatal islets could have a connection to NTPDase3 function, providing a possible mechanistic role for this gene during functional maturation (Bartley et al., 2019; Barsby and Otonkoski, 2022). This suggests that NTPDase3 may represent a genuine beta cell maturation marker in normal human development and directly play a role in beta cell function. However, NTPDase3 is expressed in fetal and neonatal acinar cells, but absent in fetal and neonatal islets; postnatally this expression shifts to be mainly beta cell-specific in adulthood, with a small number of delta cells also expressing the marker (Saunders et al., 2019) (Figure 5H). Understanding these expression patterns is relevant for the use of this marker to sort mature cells from native tissues or differentiated SC-islets.

IAPP is stored in the same vesicles as, and co-secreted with, insulin. Upon glucose stimulation, serum IAPP concentrations mirror that of insulin, and IAPP has a paracrine function in inhibiting both insulin and glucagon secretion (Westermark et al., 2011). IAPP in humans can form pathologic amylin deposits associated with the progression of T2D (Xu et al., 2021); however, mouse IAPP is not capable of forming these plaques - another example of major differences between mouse and human islet biology. Due to the role IAPP has in feedback inhibition of islet endocrine function, and because IAPP has been found to increase in beta cell differentiation along a pseudo-time scale (Veres et al., 2019), IAPP has been suggested as a marker of beta cell maturation. Our results reveal that indeed, IAPP expression significantly increases from human fetal to adult beta cells (Figure 5B) and is critically deficient in our SC-islets (Supplemental Figure S1) which lack function. The same secretory vesicles require Zn^2+^ for proper formation, maturation, and function, which relies on the islet-specific zinc transporter ZNT8, encoded by the gene *SLC30A8* (Wijesekara et al., 2009). Our results indicate that ZNT8 is expressed at about equal levels in fetal and adult human islets (Supplemental Figure S4B). Interestingly, Zn^2+^ transport plays a role in the stability and solubility of IAPP, where ZNT8 dysfunction can lead to IAPP amyloid deposition (Xu et al., 2021). It is possible that ZNT8 expression precedes IAPP expression in order to provide the appropriate environment for healthy IAPP function, and this may an important consideration for supporting healthy function of SC-islets.

Chromogranin A (CHGA) and chromogranin B (CHGB) are present in dense core secretory granules of many endocrine cell types. CHGA expression is present early in the development of pancreatic endocrine cells (Supplemental Figure S4A) (Talchai et al., 2012; Moin et al., 2018; Ramond et al., 2018). CHGB has been shown to regulate beta cell secretory granule trafficking, and loss of CHGB impairs GSIS and proinsulin processing (Bearrows et al., 2019). We now show that CHGB expression at the RNA and protein level significantly increases in the endocrine cells adult human islets compared to fetal human islets (Figure 4; Figures Fig, 5A). Another marker that has an increased expression profile at both the RNA and protein level in adult compared to fetal islets in our study is FAM159B (Figure 4; Figure 5C), which has an unknown role in islet biology, but has recently been shown to be upregulated in correlation with functional maturation in SC-islets (Augsornworawat et al., 2020; Maxwell et al., 2022).

A subset of proposed markers of beta cell maturation have direct roles in beta cell glucose uptake and metabolism. GLUT1 and GLUT2 (transcribed from *SLC2A1* and *SLC2A2*, respectively) are required for glucose transport into islet cells, and have established differences in expression between human islets, in which GLUT1 is the primary driver of glucose transport, and rodent islets in which GLUT2 is the main glucose transporter (Berger and Zdzieblo, 2020). Glucokinase (*GCK*) plays a central role in glucose processing prior to glycolysis and is considered a key factor in islet glucose sensing (Matschinsky and Wilson, 2019). Glucose-6-phosphatase catalytic subunit 2 (*G6PC2*) is a negative inhibitor of GSIS and has been found to have reduced levels in T2D samples compared to non-diabetic islets, and increased expression in SC-islets with improved function, implicating this gene in maturation (Pound et al., 2013; Barsby and Otonkoski, 2022). We find that *GCK* expression does not change between human fetal and adult islets (Figure 4). G6PC2 expression at the gene and protein level, however, increases from fetal to adult stages (Figure 4) (Figure 5D), and increases following *in vivo* HFP maturation (Figures 6B–E). GLUT1 is very highly expressed in undifferentiated stem cells, maintains elevated expression throughout SC-islet differentiation (Supplemental Figure S7), and is also not islet-specific; Glut1 is expressed in other pancreas cells in both fetal and adult human pancreas (Figure 5E). These variables can make islet-specific GLUT1 expression at the RNA and protein level difficult to assess. However, quantification of immunofluorescent staining indicates that GLUT1 expression is significantly increased in correlation with beta cell maturation (Figure 5E) (Figures 6D,E).

It is still not well understood how transplantation of cells and tissues affects engrafted cell maturation. Once SC-islets are transplanted into a suitable environment, they experience myriad interactions and signals that are absent *in vitro*, including vascularization, frequent stimulation by feeding cycles, circadian entrainment, contact with the *in vivo* microenvironment including ECM-signaling and cell-cell interactions with endothelial, mural, neuronal, and immune cells. The effects that some of these individual elements have on islet and SC-islet health and function have been explored *in vitro* through many different studies, and each have been shown to have influence on the identity and function of the cells (Alvarez-Dominguez et al., 2020; Singh et al., 2020; Nguyen-Ngoc et al., 2022; Tremmel et al., 2022). Together, it is possible that these elements of the tissue microenvironment provide combined cues to stimulate the final push towards maturation that cells require. Combining some of these elements with SC-islets *in vitro* may help improve SC-islet maturation profiles, and better understanding markers of maturation will help to assess the outcomes of this effort.

In summary, understanding the normal expression profiles of potential maturation markers in human islets is essential to generating functional and safe SC-islets. While certain markers that we have identified do not have significant differences in expression between fetal and adult islets (*ERO1LB, HDAC9, GCK, GLUT2, UCN3, ZNT8*), it is notable that the expression of many of these markers remain deficient in the SC-islets derived in this study (Supplemental Figure S1, Supplemental Figure S7), and therefore merit further attention to ensure proper expression is achieved in future protocols. Markers with significantly different expression between adult and fetal beta cells may have applications to help improve identification of mature beta cells and possibly to unravel roles these genes play in the regulation of glucose-sensitive insulin secretion. Furthermore, many of these markers have been found to change in expression with the progression of diabetes, and thus, a better understanding of the roles of these maturation-associated genes in normal islet function may inform future therapies to prevent or treat diabetes.

## Methods

### Tissue procurement and ethics

Within 24 h of recovery, the organs were received and cleaned of surrounding connective tissue. Immediately upon receipt, tissue was used for transplantation studies or preserved for further analyses. Small pieces of tissue were fixed with 4% PFA for paraffin embedding, or equilibrated in 30% sucrose and OCT-embedded. Other pieces were immediately lysed in TRIzol (ThermoFisher, 15596026) for RNA extraction. Two small pieces of tissue (ranging from 5–10 mg in weight) from each donor were also immediately washed and used for functional studies (GSIS).

Adult human pancreas tissue was procured by the University of Wisconsin Organ and Tissue Donation Services from donors with no indication of diabetes or pancreatitis, with consent obtained for research from next of kin and authorization by the University of Wisconsin-Madison Health Sciences Institutional Review Board (IRB granted an exempt from protocol approval for studies on post-natal tissue because research on deceased donors is not considered human subjects research). IRB oversight of the project is not required because it does not involve human subjects as recognized by 45 CFR 46.102(f) which defines a ‘human subject’ as “a living individual about whom an investigator (whether professional or student) conducting research obtains (1) data through intervention or interaction with the individual, or (2) identifiable private information.” Following organ recovery, pancreata were allocated for research if deemed unfit for transplantation due to vascular damage during organ recovery, no suitable recipient, and non-ideal age or BMI. The organs were received within 24 h of recovery and trimmed of extra-pancreatic connective tissues, including duodenum, large arteries and veins. The parenchyma was cut into 1 cm^3^ cubes and fixed with 4% PFA for paraffin embedding, or equilibrated in 30% sucrose and OCT-embedded.

All donor tissues used are summarized in Supplemental Figure S4.

### Human islets

Human islets were received through the Integrated Islet Distribution Program (IIDP) and experiments were performed within 48 h of receipt. Islets used for QPCR were lysed with TRIzol (ThermoFisher, 15596026) for gene expression analysis.

All human islets used in this study are summarized in Supplemental Figure S4.

### Cell culture and differentiation

H1 (WA01, WiCell) cells were differentiated toward a pancreatic endocrine fate using protocols based on Xu et al. (2011), Rezania et al. (2014), Pagliuca et al. (2014), Nostro et al. (2015) and Zhu et al. (2016). Media components for each stage of differentiation for both Protocol A and Protocol B are included in detail in Supplemental Tables S1–3. To initiate the differentiation, H1 colonies were treated with ROCK-Inhibitor for 4 h (Y-27632) and plated as single cells onto Matrigel-coated 6-well Transwell plates (Corning, 3450) at a density of 1.5 × 10 (Balboa et al., 2022) cells per cm^2^ surface area. Standard E8 medium (ThermoFisher, A1517001) was added to both sides of the Transwell to expand the cells before differentiation was initiated (1–2 days). When the cells covered the Transwell membranes at near 100% confluence, Stage 1 was initiated, this was considered “Day 0”. Full medium changes were made every day thereafter until the end of Stage 4, in accordance with Supplemental Tables S1, 2. At the end of Stage 4, cells were transferred into suspension as follows: adherent cells were treated with Versene for 5 min at 37°C, the Versene was removed and the sheet of cells was pipetted with Stage 4 medium until the cell sheets were broken into small pieces (∼500–1000 microns in diameter). The cells were pelleted, changed into Stage 5 medium, and plated into Ultra Low Attachment (ULA) plates in stationary culture for the remainder of the differentiation. The next day, the cells will appear spherical in shape with an average diameter of 200–250 microns. During Stage 5, media was changed 50% each day. In Stages 6, 7 and 8, media was fully changed every other day, as indicated in Supplemental Tables S1–3.

### Glucose-stimulated insulin secretion (GSIS)

Static GSIS assays were performed in series, in 24-well plates with cell filter inserts (PIXP01250, MilliporeSigma, St. Louis, MO). Cells were added to the filters and moved from low glucose (2.8 mM) to high glucose (28 mM) to low glucose (2.8 mM) to a depolarization solution (30 mM KCl, 2.8 mM glucose). For HFP tissues, 5–10 mg of fresh tissue was added to each well for GSIS. All solutions for GSIS were made in Krebs buffer (25 mM HEPES, 115 mM NaCl, 24 mM NaHCO3, 5 mM KCl, 1 mM MgCl2, 2.5 mM CaCl2, 0.1% BSA). The supernatant was collected following 1 h in each step of the GSIS for secreted C-pep measurement. For human islets, 100 IEQ were used per well. For differentiated SC-islets, 500 IEQ were used per well. Supernatants collected from each treatment were frozen in aliquots until analysis.

For HFP tissue and primary islets, following GSIS, cells were lysed in 1 mL of lysis buffer (20 mM Tris-HCl, pH 7.5, 150 mM NaCl, 1 mM EDTA, 1% Triton) and homogenized with a PowerGen 500 homogenizer (ThermoFisher, Waltham, MA); these lysates were used to measure total C-peptide content. C-peptide content for all lysates and supernatants were determined with an ultra-sensitive human C-pep ELISA (Mercodia, Uppsala, Sweden). Stimulation index (SI) for the static GSIS was calculated by dividing the average secreted C-Peptide concentration under high glucose by the average C-Peptide secreted under the first low glucose period.

### Transplantation

NOD-scid IL2rγ^null^ (NSG) mice, 8–12 weeks of age, were made diabetic with a single 150 mg/kg intraperitoneal (IP) dose of streptozotocin (STZ). Following a measurable increase in blood glucose above 400 mg/dL and systemic clearance of the STZ, each mouse was transplanted with a ∼3 mm^3^ piece of HFP (17–20 gestational week age range at time of procurement) into the kidney subcapsular (KSC) space. Non-fasted blood glucose levels were monitored weekly and IP-GTTs were performed at weeks 12, 25 and 32 post-transplantation.

### Glucose tolerance test (GTT)

At 12, 25, and 32 weeks, mice were fasted overnight prior to the glucose tolerance test (GTT). A 30% D-glucose IP injection at a dose of 2 g/kg initiated the GTT at 0’. Blood glucose was measured at time 0’ (fasted, before injection), 15′, 30′, 60′, and 120′ thereafter. Blood was collected at time 0’ (fasted, before injection) and at 15’ post-injection to determine serum C-Pep. Serum C-pep was measured using an ultra-sensitive human C-peptide ELISA kit (Mercodia, Uppsala, Sweden).

### Immunofluorescent (IF) staining

For most antibodies, formalin-fixed paraffin embedded (FFPE) sections were used. 5 µm paraffin sections were deparaffinized using xylene and rehydrated. Antigen retrieval was performed by treatment with 10 mM Citrate Buffer, pH6.0 for 2 h in an 80°C water bath. Slides were blocked with 10% BSA/1x PBS for 1 h at room temperature, incubated with primary antibodies overnight at 4°C, washed, incubated with secondary antibodies for 40 min at room temperature.

For antibodies that did not react with FFPE sections (NTPDase3), frozen sections were used. 5 µm sections were cut from unfixed OCT-embedded tissues, warmed to room temperature for 15 min and fixed in 4% PFA for 15 min. Slides were then washed, blocked and stained following the same steps as FFPE sections.

All antibodies and dilutions used are listed in Supplemental Table S5. For some antibodies (indicated with an asterisk in Supplemental Table S5), signal was amplified using Tyramide SuperBoost™ Kits (Rabbit Alexa Fluor 488, B40922, Invitrogen) (Mouse Alexa Fluor 594, B40942, Invitrogen), following manufacturer protocol. Nuclei were counterstained with 40-6-diamidino-2-phenylindole (DAPI). 20x and 40x images were collected using a Zeiss Axiovert 200M microscope.

### QPCR

All samples were lysed in TRIzol (ThermoFisher, 15596026) and RNA was extracted using the QIAprep Spin Miniprep kit (QIAGEN, 27,104). cDNA was prepared from extracted RNA using the Omniscript RT kit (QIAGEN, 205113). Quantitative Real-Time Polymerase Chain Reaction (QPCR) was performed using the TaqMan Real-Time PCR Master Mix (ThermoFisher, 4304437) and Taqman Gene Expression Assay Primers (ThermoFisher) listed in Supplemental Table S6.

### Image quantification

Images were quantified using ImageJ. Quantification was performed by two individuals blinded to the sample identify, and automated quantification methods were used to keep the analysis as unbiased as possible. For co-localization measurements, binary images were generated for each color channel and the JACoP plugin (Bolte and Cordelieres, 2006) was used and Manders coefficients quantified. For proteins that localize to the nucleus, total nuclei were counted using Measure Particles in ImageJ on the binary DAPI channel and individual nuclei were counted in Ins^+^ or Ins^−^ cells to determine the percentage of positive cells for each group. For each analysis, at least 10 islets were imaged and quantified per donor, and three to five donors were used per group.

### Statistical analyses

Data are reported as average ±standard deviation unless otherwise indicated. All *p*-values were calculated with a Student’s two-tailed *t*-test using Prism six for Windows (GraphPad). Each sample set was evaluated for normality using the Shapiro-Wilk test (alpha = 0.05) prior to performing Student’s t-test. Prism’s suggested significance classification scheme was followed (*p < .05) (**p < .01) (***p < 0.001) (****p < 0.0001).

## Data Availability

The raw data supporting the conclusion of this article will be made available by the authors, without undue reservation.
