# Supplementary material for: Validating expression of beta cell maturation-associated genes in human pancreas development
Source: Front Cell Dev Biol. 2023 Feb 1;11:1103719. doi: 10.3389/fcell.2023.1103719 (PMC9945361; doi:10.3389/fcell.2023.1103719)
Supplement: Supplementary file 1 [file DataSheet1.pdf]

Supplemental Information

Validating expression of beta cell maturation-associated genes in human pancreas development

Tremmel et al.

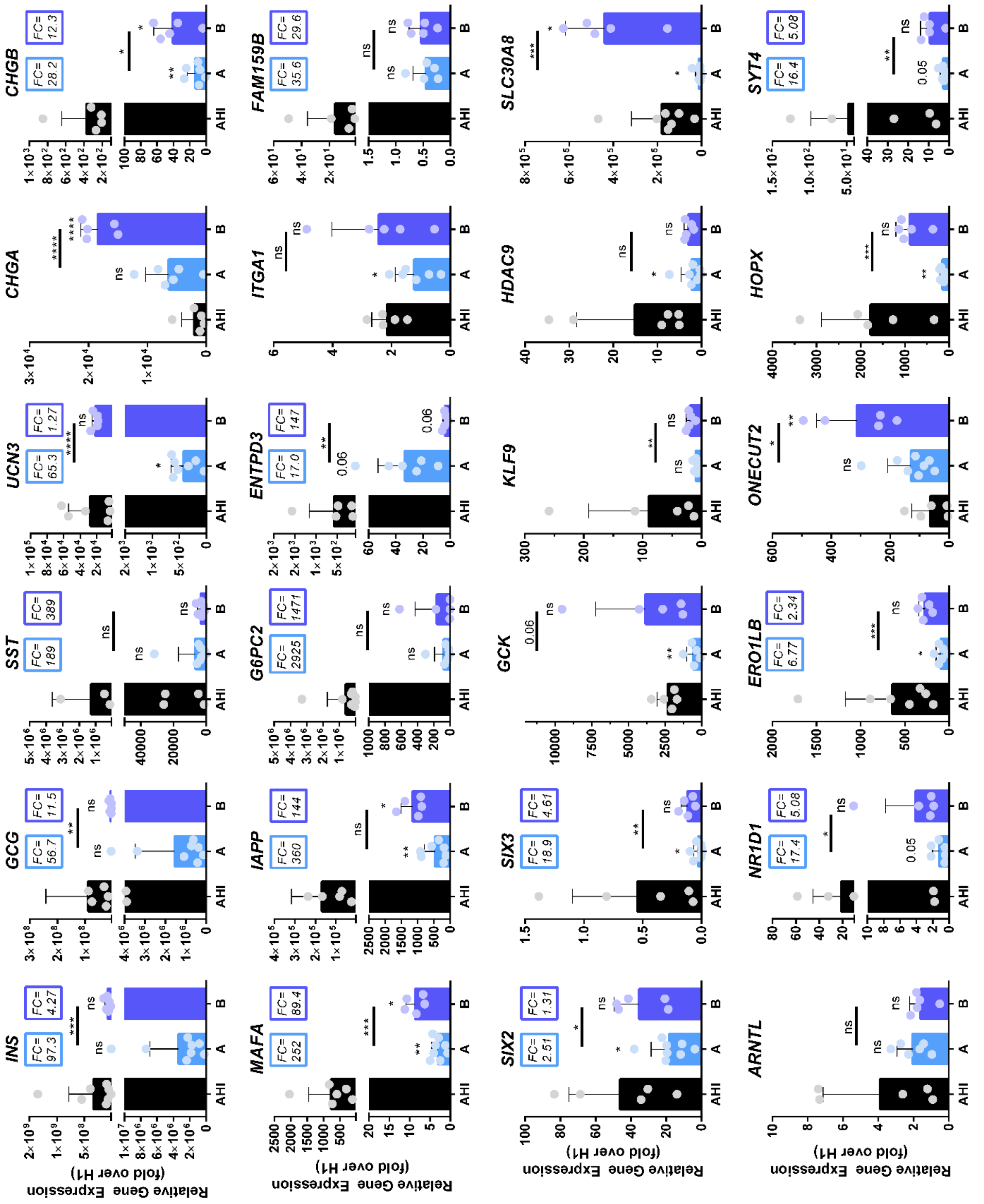
INS
GCG
SST
UCN3
CHGA
CHGB
MAFA
IAPP
G6PC2
ENTPD3
ITGA1
FAM159B
SIX2
SIX3
GCK
KLF9
HDAC9
SLC30A8
ARNTL
NR1D1
ERO1LB
ONECUT2
HOPX
SYT4
Relative Gene Expression (fold over H1)

Supplemental Fig. 1 Gene expression of SC-islets derived from Protocols A and B

1A) Gene expression determined by QPCR of endocrine genes and maturation-associated genes in SC-islets derived from Protocols A (N=5-8) and B (N=5-6), compared to primary adult human islets (AHI)(N=5-7). All genes were normalized to β actin within the same sample, then normalized to undifferentiated H1 cells. Fold changes (FC) listed are calculated based on the relative expression between AHI and each protocol. **(Previous Page)**

1B) Gene expression determined by QPCR of endocrine genes and maturation-associated genes in SC-islets derived from Protocols A (N=5-8) and B (N=5-6), compared to primary adult human islets (AHI)(N=5-7) and human fetal pancreas (HFP)(N=5). All genes were normalized to β actin within the same sample, then normalized to undifferentiated H1 cells. To compare gene expression profiles between endocrine cells among the four samples, all samples were then normalized to CHGA within the same sample. Fold changes (FC) listed are calculated based on the relative expression between AHI and each protocol. **(Next Page)**

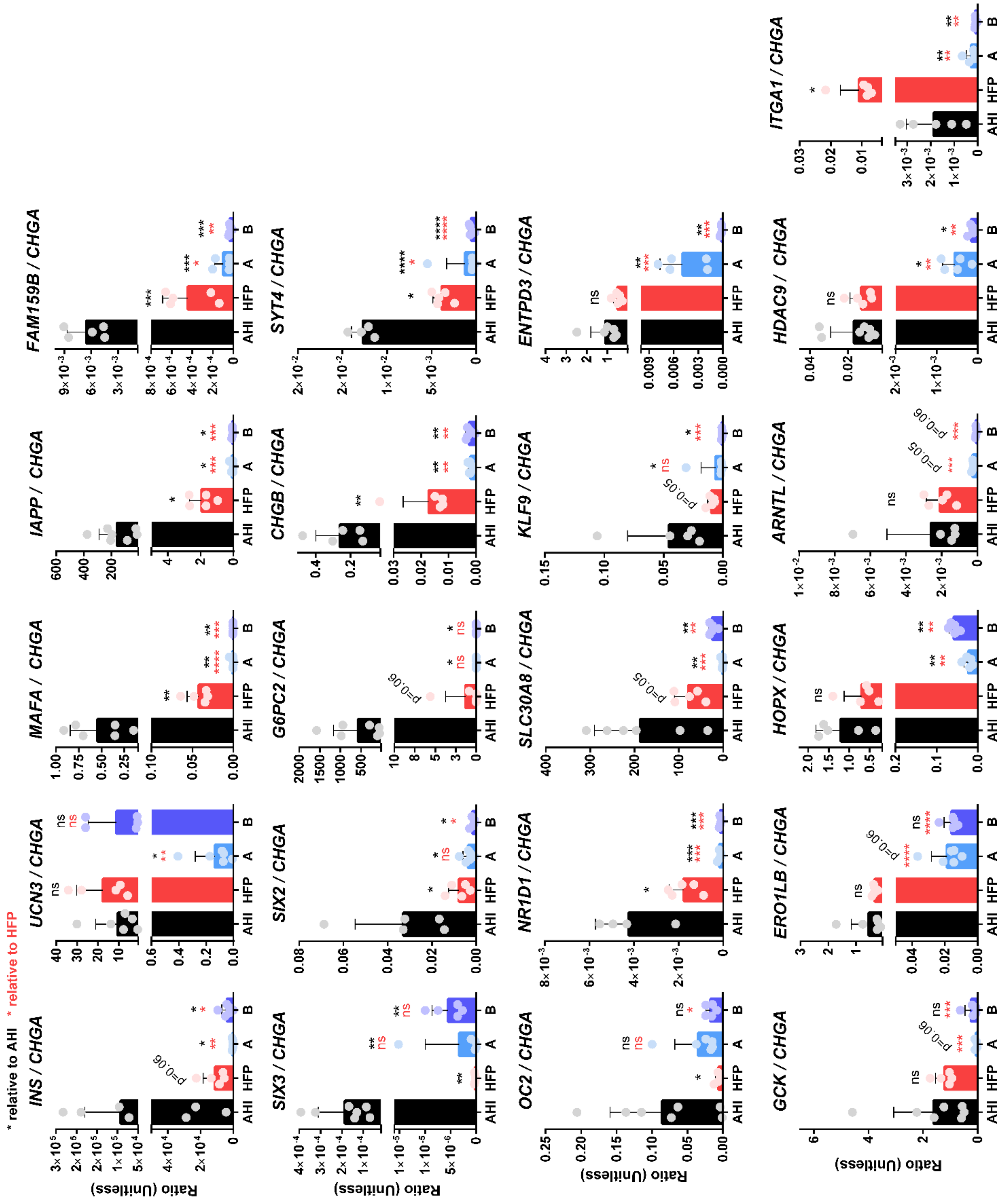
* relative to AHI
* relative to HFP
Ratio (Unitless)
INS / CHGA
UCN3 / CHGA
MAFA / CHGA
IAPP / CHGA
FAM159B / CHGA
SIX3 / CHGA
SIX2 / CHGA
G6PC2 / CHGA
CHGB / CHGA
SYT4 / CHGA
OC2 / CHGA
NR1D1 / CHGA
SLC30A8 / CHGA
KLF9 / CHGA
ENTPD3 / CHGA
GCK / CHGA
ERO1LB / CHGA
HOPX / CHGA
ARNTL / CHGA
HDAC9 / CHGA
ITGA1 / CHGA
AHI
HFP
A
B
ns
p=0.06
p=0.05

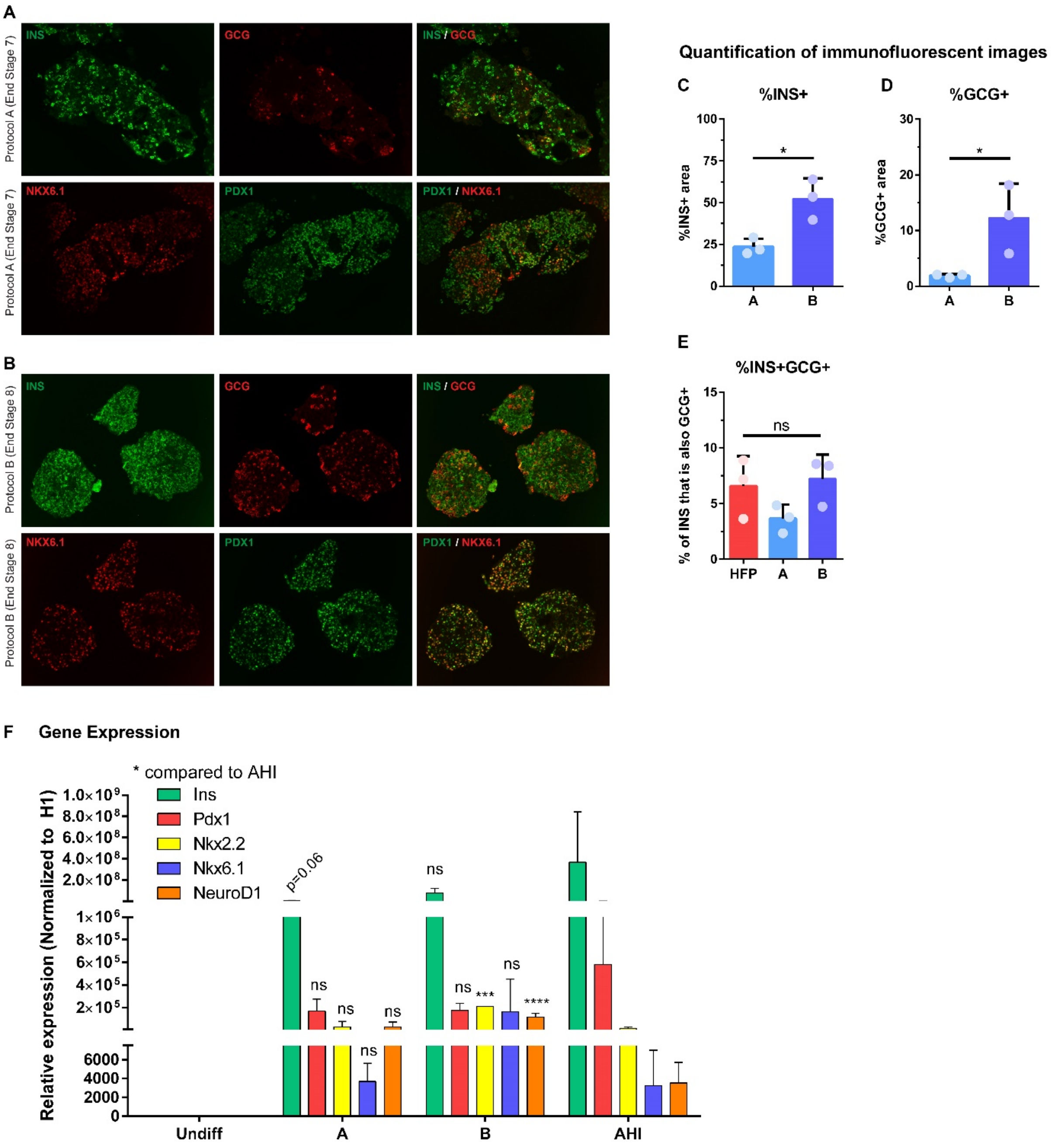

Supplemental Fig. 2 | Assessment of SC-islets generated in Protocols A and B
Immunofluorescent staining for insulin (green, top), glucagon (red, top), Nkx6.1 (red, bottom) and Pdx1 (green, bottom) for SC-islets from Protocol A **(A)** and Protocol B **(B)**. Quantification of the percentage of cell area that is positive for insulin **(C)** and glucagon **(D)** in SC-islets dervided from Protocols A and B, and the percentage of insulin-positive area that is double positive for glucagon in SC-islets from Protocols A and B, and human fetal pancreas (HFP) **(E)**. QPCR gene expression data for characteristic islet genes in undifferentiated cells (H1 cells, normalized to 1 for each gene), Protocol A, Protocol B and adult human islets (AHI) **(F)**. Statistical comparisons are relative to AHI.

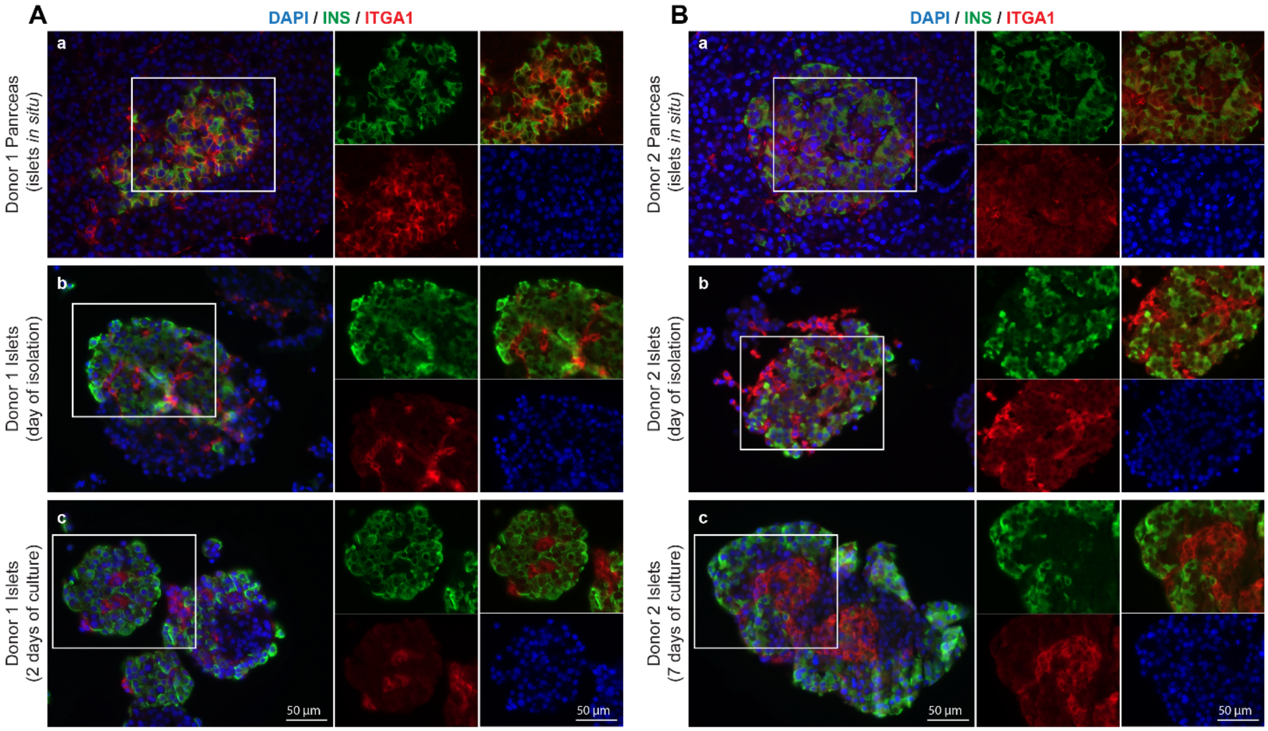

Supplemental Fig. 3 | ITGA1 immunostaining in human islets in situ, during isolation, and during culture

Immunofluorescent staining of ITGA1 in islets at different stages of isolation and culture, from two different islet isolations (A, Donor A and B, Donor B). **(A)** Islets from donor A were stained before isolation (*in situ*) (a), collected during isolation (b), and collected after 2 days of culture (c). **(B)** Islets from donor B were stained before isolation (*in situ*) (a), collected during isolation (b), and collected after 7 days of culture (c).

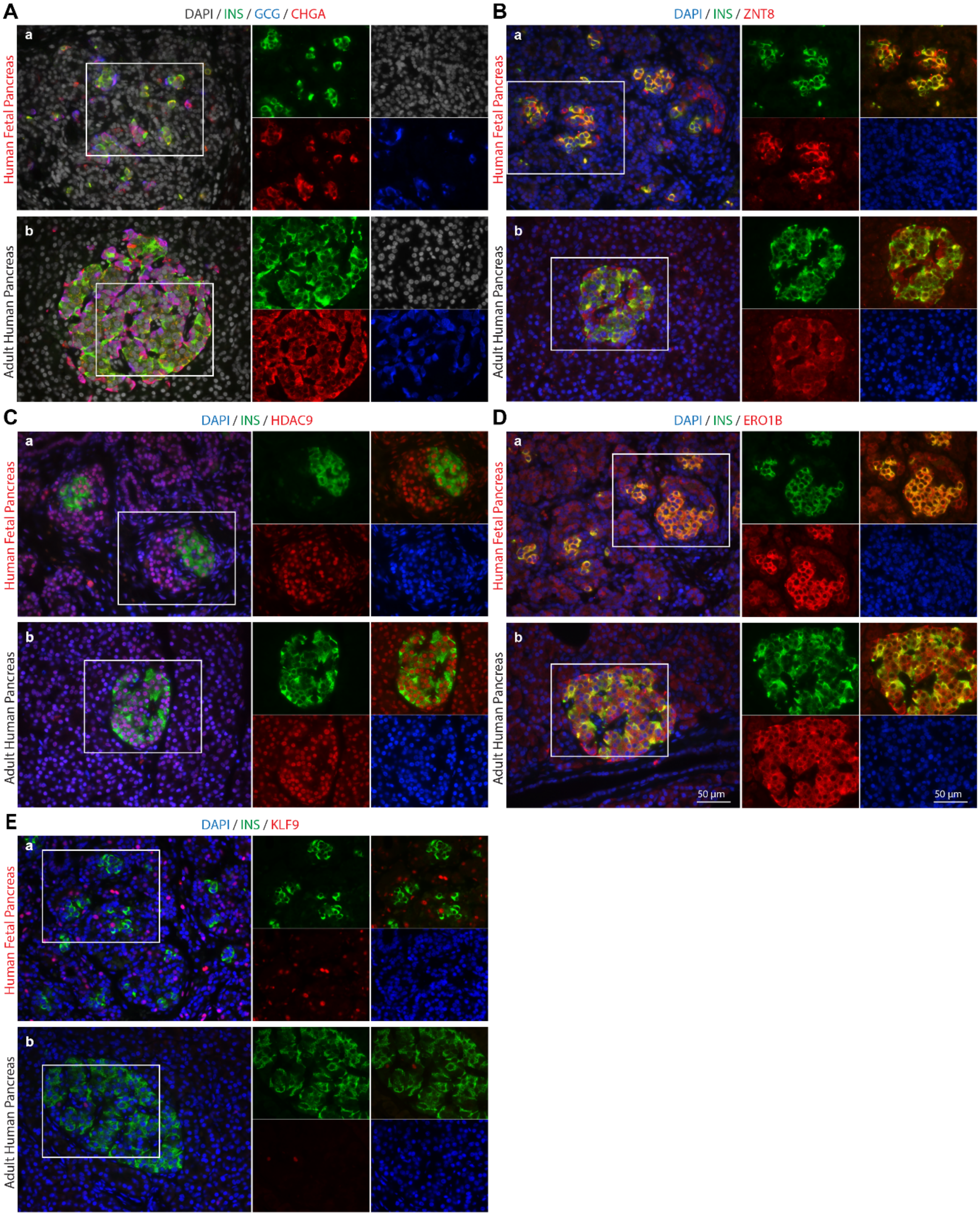

Supplemental Fig. 4 | Additional immunofluorescent staining of fetal and adult human islets

Immunofluorescent staining of candidate human beta cell maturation markers reveals no change in beta cell-specific expression of the markers CHGA **(A)**, ZNT8 **(B)**, HDAC9 **(C)**, ERO1B **(D)**, KLF9 **(E)** between human fetal and adult pancreas sections. Individual channel images are the same magnification as the larger merged images. Scale bars = 50 microns.

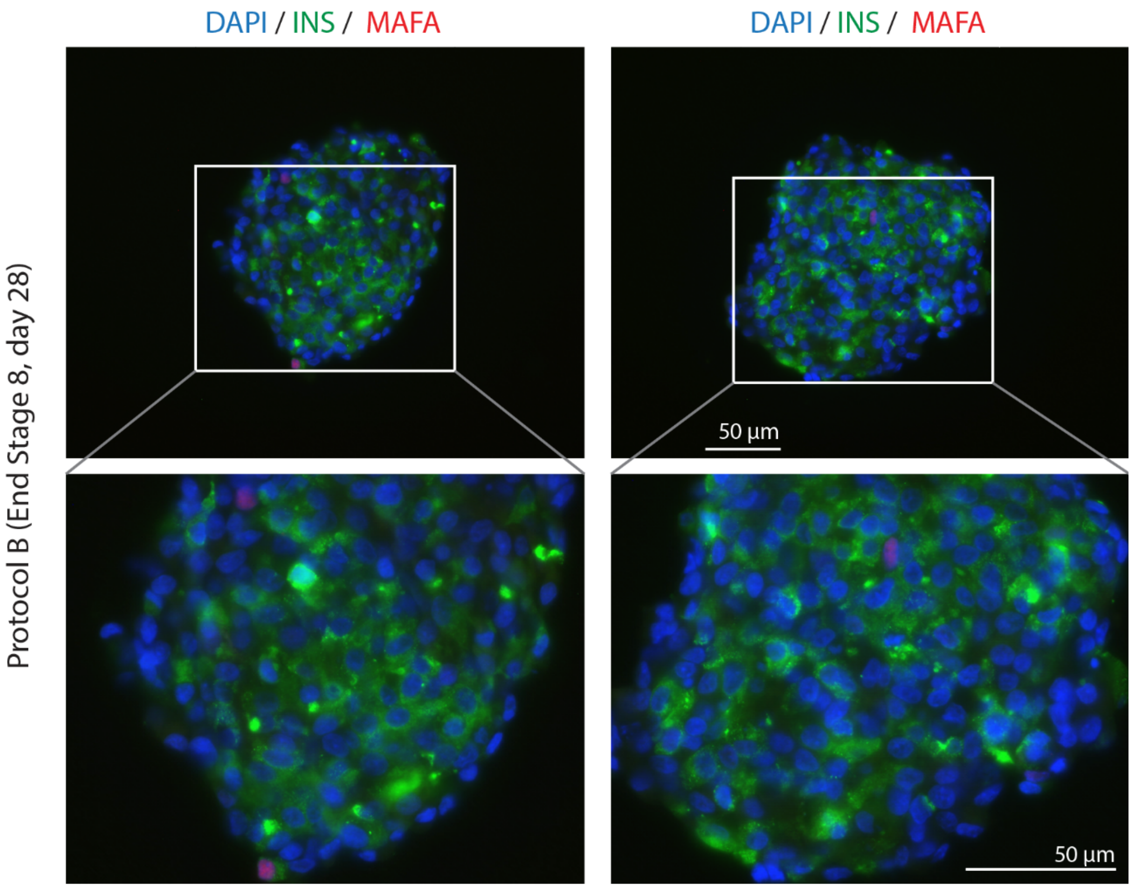

Supplemental Fig. 5 | MAFA expression is low in Protocol B SC-islets.

To validate the accuracy of the MAFA antibody and our findings that MAFA is expressed in human fetal beta cells, Protocol B SC-islets were stained for MAFA (red), demonstrating very low MAFA protein expression which correlates with gene expression data for these cells.

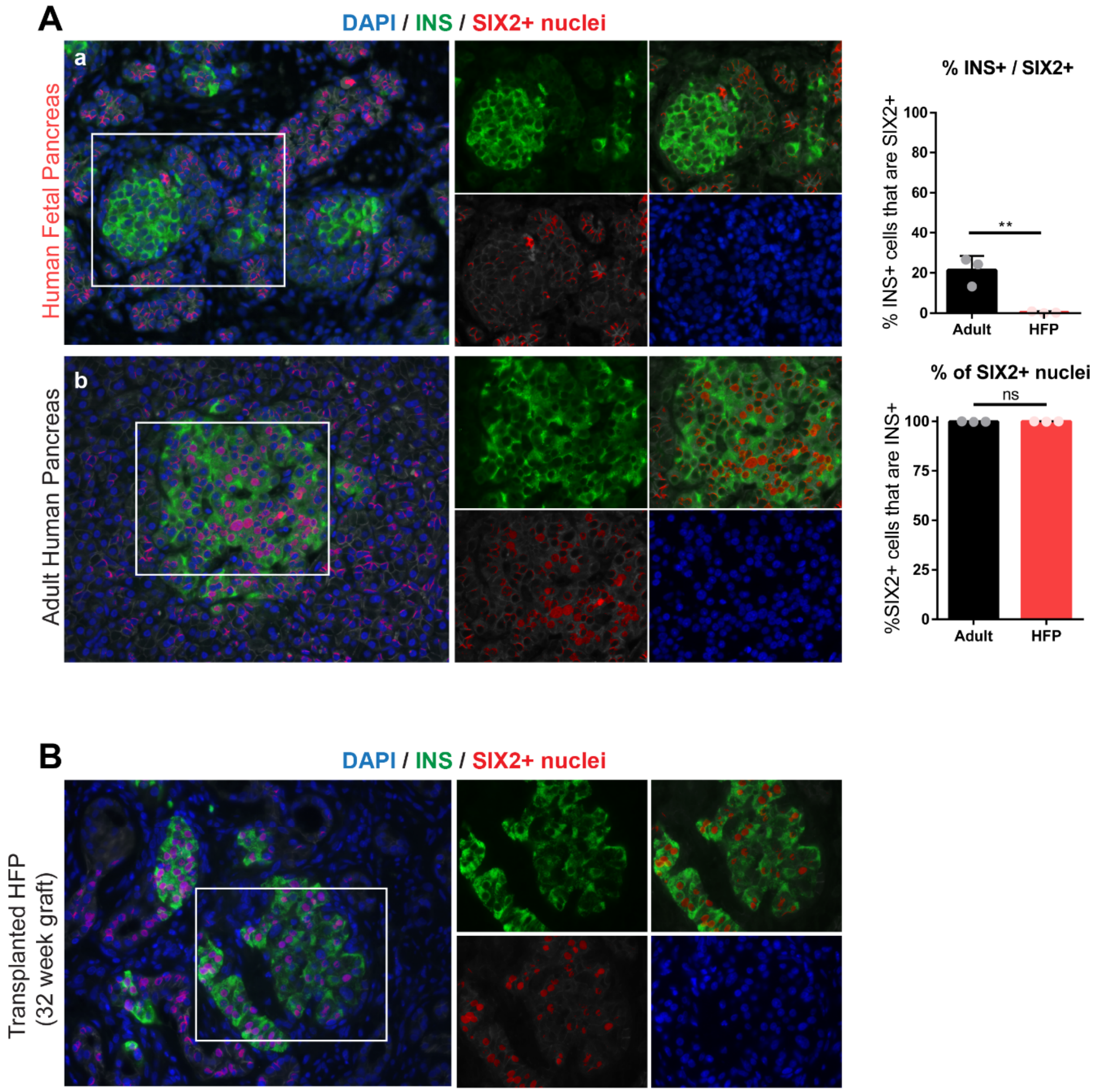

Supplemental Fig. 6 | Nuclear SIX2 expression correlates with beta cell maturation.

Nuclear expression of SIX2 is low in human fetal beta cells and significantly increased in adult beta cells **(A)**. Nuclear expression of SIX2 is high in HFP grafts after an additional 32 weeks of *in vivo*-maturation **(B)**. Representative images (N=3 donors per group).

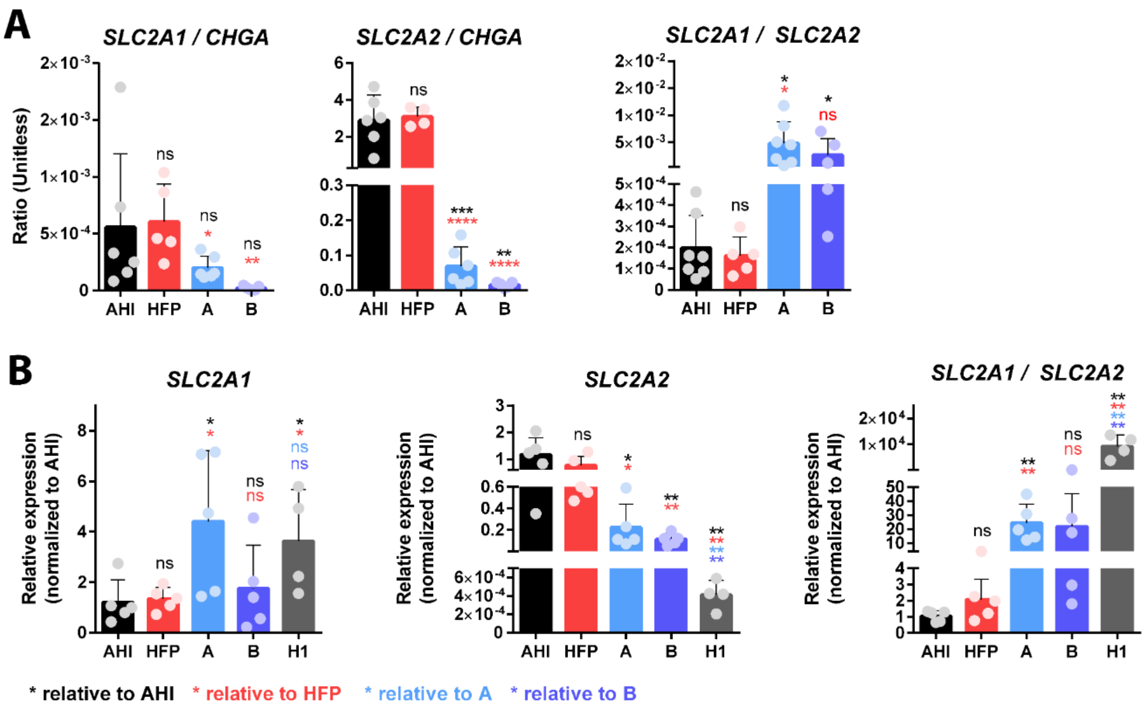

Supplemental Fig. 7 | Expression of glucose transporters GLUT1 and GLUT2

QPCR-based analysis of gene expression of glucose transporters *SLC2A1* (GLUT1) and *SLC2A2* (GLUT2) in adult human islets ("AHI"), human fetal pancreas ("HFP") and Protocols A ("A") and B ("B"). **A)** Gene expression was normalized to beta-actin within the same sample, then normalized to undifferentiated H1 cells and presented as a ratio of gene expression over *CHGA* to normalize for differences in endocrine mass between the various samples. **B)** Gene expression was normalized to beta-actin within the same sample, then normalized to AHI. Statistics relative to AHI are represented in black font, relative to HFP are represented in red font, relative to Protocol A are represented in light blue font, and relative to Protocol B are represented in purple font.

Supplemental Table 1: Protocol A

	Stage	Day	Media		Supplement								
Adherant	S0 (2d)	**d-2**	seeded 750,000 cells/well of a 6-well Transwell plate (Corning, 3450)										
		**d-1**											
	S1 (3d)	**d0**	Begin differentiation when cells reach ~90% confluency										
		**d0**	***Basal A1***		**CHIR-99021**<br>2uM	**Activin A**<br>100ng/ml	**bFGF**<br>0.1 ug/ml	**BMP4**<br>0.01ug/ml					
		**d1**	***Basal A1***		**CHIR-99021**<br>0.2uM	**Activin A**<br>100ng/ml	**bFGF**<br>0.1 ug/ml	**BMP4**<br>0.01ug/ml					
		**d2**	***Basal A1***			**Activin A**<br>100ng/ml	**bFGF**<br>0.1 ug/ml	**BMP4**<br>0.01ug/ml					
	S2 (2d)	**d3**	***Basal A1***		**Vitamin C**<br>0.25mM	**FGF7**<br>50ng/ml	**RA**<br>0.5uM	**LDN**<br>100ng/ml	**SB-431542**<br>2.5mM				
		**d4**	***Basal A1***		**Vitamin C**<br>0.25mM	**FGF7**<br>50ng/ml	**RA**<br>0.5uM	**LDN**<br>100ng/ml	-				
	S3 (3d)	**d5 - 7**	***Basal A2***		**Vitamin C**<br>0.25mM	**FGF7**<br>50ng/ml	**RA**<br>1.0uM	**LDN**<br>200ng/ml	**Sant-1**<br>0.25uM	**TPB**<br>0.2uM	**ITS-X**<br>0.5 x		
	S4 (3d)	**d8 - 10**	***Basal A2***		**Vitamin C**<br>0.25mM	**FGF7** 2ng/ml	**RA**<br>0.1uM	**LDN**<br>100ng/ml	**Forskolin**<br>10uM	**SANT-1**<br>0.25 uM	**ITS-X**<br>0.5 x		
	S5 (3d)		On day 11: S4 cells are treated with 10um Y-27632 for 4 hours, rinsed with PBS -/- and treated with versene for 3-5 mins. Keep cells in clusters, making sure they are not too small. Spin 300xg for spin. Gently resuspend pellet in stage 5 medium - keeping attention to size of clusters, try to keep ~100um. Plate cells into ultra-low attachment plates or T25s										
Suspension		**d11 - 13**<br>*50% change each day*	***Basal A3***		**Nico**<br>10mM	**ITS-X**<br>0.5 x	**Alk5i II**<br>0.01mM	**Heparin**<br>10ug/ml	**T3**<br>1.0uM	**LDN**<br>100ng/ml	**Forskolin**<br>10uM	**Exendin-4**<br>0.01uM	**RA**<br>0.025 uM
	S6 (7d)	**d14 - 20**<br>*Feed every other day*	***Basal A3***		**Nico**<br>10mM	**ITS-X**<br>0.5 x	**Alk5i II**<br>0.01mM	**Heparin**<br>10ug/ml	**T3**<br>1.0uM	**LDN**<br>100ng/ml	**Forskolin**<br>10uM	**Exendin-4**<br>0.01uM	**DAPT**<br>1 uM
	S7 (7d)	**d21 - 27**<br>*Feed every other day*	***Basal A3***		**Nico**<br>10mM	**ITS-X**<br>0.5 x	**Alk5i II**<br>0.01mM	**Heparin**<br>10ug/ml	**T3**<br>1.0uM	**BMP4**<br>0.01ug/ml	**NAC**<br>1mM	**Trolox**<br>10uM	**R428**<br>2uM

Supplemental Table 2: Protocol B

	Stage	Day	Media	Supplement									
Adherant	S0 (2d)	**d-2**	seeded 750,000 cells/well of a 6-well Transwell plate (Corning, 3450)										
		**d-1**											
	S1 (3d)	**d0**	Begin differentiation when cells reach ~90% confluency										
		**d0**	***Basal B1***	**CHIR-99021**<br>2uM	**Activin A**<br>100ng/ml								
		**d1**	***Basal B1***		**Activin A**<br>100ng/ml	**bFGF**<br>0.005 ug/ml							
		**d2**	***Basal B1***		**Activin A**<br>100ng/ml	**bFGF**<br>0.005 ug/ml							
	S2 (3d)	**d3 - 5**	***Basal B2***	**Vitamin C**<br>0.50mM	**FGF10**<br>50ng/ml	**CHIR-99021**<br>2uM	**LDN**<br>100ng/ml						
	S3 (2d)	**d6 - 7**	***Basal B3***	**Vitamin C**<br>0.285mM	**FGF10**<br>50ng/ml	**RA**<br>2.0uM	**LDN**<br>100ng/ml	**B27 (-VitA)**<br>1%					
	S4 (3d)	**d8 - 10**	***Basal B3***	**Vitamin C**<br>0.285mM	**EGF**<br>100ng/ml	**PDBu**<br>0.03uM	**LDN**<br>100ng/ml	**B27**<br>1%	**Nico**<br>10mM				
	S5 (3d)		On day 11: S4 cells are treated with 10um Y-27632 for 4 hours, rinsed with PBS -/- and treated with versene for 3-5 mins. Keep cells in clusters, making sure they are not too small. Spin 300xg for spin. Gently resuspend pellet in stage 5 medium - keeping attention to size of clusters, try to keep ~100um. Plate cells into ultra-low attachment plates or T25s										
Suspension		**d11 - 13**<br>*50% change each day*	***Basal B4***	**Vitamin C**<br>0.25mM	**LDN**<br>100ng/ml	**Alk5i II**<br>0.01mM	**Heparin**<br>10ug/ml	**Comp E**<br>1.0uM	**Nico**<br>10mM	**Forskolin**<br>10uM	**Exendin-4**<br>0.01uM	**RA**<br>0.025 uM	
	S6 (4d)	**d14 - 17**<br>*Feed every other day*	***Basal B4***	**Vitamin C**<br>0.25mM	**LDN**<br>100ng/ml	**Alk5i II**<br>0.01mM	**Heparin**<br>10ug/ml	**Comp E**<br>1.0uM	**Nico**<br>10mM	**T3**<br>1.0uM	**$ZnSO_4$**<br>10uM	**BayK**<br>2uM	
	S7 (7d)	**d18 - 21**<br>*Feed every other day*	***Basal B4***	**Vitamin C**<br>0.25mM	**BMP4**<br>0.01ug/ml	**Alk5i II**<br>0.01mM	**Heparin**<br>10ug/ml	**R428**<br>2uM	**NAC**<br>1.0mM	**T3**<br>1.0uM	**$ZnSO_4$**<br>10uM	**BayK**<br>2uM	**Trolox**<br>10uM
	S8 (7d)	**d21 - 27**<br>*Feed every other day*	***Basal B5***	**MK-801**<br>10uM	**T3**<br>1.0uM	**Alk5i II**<br>0.01mM							

Supplemental Table 3: Basal Media

**Basal Media:**							
Basal A1	MCDB131	**Glutamax** 1 x	**BSA** 0.5%	**$NaHC0_3$** 1.5 g/L	**Glucose** 10mM		
Basal A2	MCDB131	**Glutamax** 1 x	**BSA** 2%	**$NaHC0_3$** 2.5 g/L	**Glucose** 10mM		
Basal A3	MCDB131	**Glutamax** 1 x	**BSA** 2%	**$NaHC0_3$** 1.5 g/L	**Glucose** 20mM	**P/S** 1 x	
Basal B1	RPMI						
Basal B2	IMDM: Ham's F12 75:25	**P/S** 1 x	**Glutamax** 1 x	**BSA** 0.05%	**N2** 0.5 x	**B27** 0.5 x	**MTG** 0.45mM
Basal B3	DMEM	**P/S** 1 x					
Basal B4	MCDB131	**P/S** 1 x	**Glutamax** 1 x	**BSA** 2%	**$NaHC0_3$** 1.5 g/L	**Glucose** 20mM	**ITS-X** 0.5X
Basal B5	CMRL supplemented	**P/S** 1 x	**AB Serum** 5%				

Supplemental Table 1: Protocol A

Protocol for differentiation under Protocol A. Regent sources and abbreviations are defined in Supplemental Table 7.

Supplemental Table 1: Protocol A

Protocol for differentiation under Protocol B. Regent sources and abbreviations are defined in Supplemental Table 7.

Supplemental Table 3: Basal Media

Recipes for basal media used in Protocols A and B. Regent sources and abbreviations are defined in Supplemental Table 7.

Supplemental Table 4: Donor Tissues

Isolated Adult Human islets

Islet Source	Islet RRID	Age	Sex	BMI	Islet Purity	Islet Viability	Cause of Death
IIDP	SAMN08768974	28 years	Male	29.2	85%	98%	Head trauma
IIDP	SAMN08768991	51 years	Male	29	80%	95%	Head trauma
IIDP	SAMN08769201	24 years	Male	34.8	95%	97%	Head trauma
IIDP	SAMN08769390	33 years	Female	34.2	95%	98%	Cerebrovascular/stroke
IIDP	SAMN08769826	30 years	Male	56.8	95%	98%	Anoxia
IIDP	SAMN12670838	40 years	Male	30.7	90%	90%	Head trauma
Internal	UWHI325R	46 years	Male	39.5	80%	98%	Cardiac Arrest

Adult Human Pancreas

Donor	Age	Sex	BMI
Donor 52	61 years	Female	25
Donor 64	22 years	Male	33.6
Donor 66	38 years	Male	23.6
Donor 68	57 years	Female	27.2
Donor 84	46 years	Male	31.9
Donor 92	42 years	Male	23.3
Donor 93	24 years	Female	24
Donor 98	35 years	Male	26.5

Human Fetal Pancreas

Donor	Age	Sex
HFP 85	18 gw	Male
HFP 88	18 gw	Unk
HFP 92	19 gw	Unk
HFP 99	17 gw	Female
HFP 102	18 gw	Male

gw = gestational week

Supplemental Table 5: Antibodies used for IF

Target	Species	Dilution	Product
CHGA	Rabbit	1:400	23342-1-AP (Proteintech)
CHGB	Rabbit	1:200	14968-1-AP (Proteintech)
ERO1LB	Rabbit	1:50	ab230540 (Abcam)
FAM159B*	Rabbit	1:50*	PA5-52855 (ThermoFisher)
GCG	Mouse	1:1000	G2654 (Sigma)
GCG	Rabbit	1:2000	ab92517 (Abcam)
G6PC2*	Rabbit	1:250*	LS-C678007 (LS Bio)
GLUT1*	Rabbit	1:250*	ab115730 (Abcam)
HDAC9	Rabbit	1:100	MA5-33151 (ThermoFisher)
IAPP	Rabbit	1:1000	ab254259 (Abcam)
INS	Mouse	1:5000	I2018 (Sigma)
INS	Guinea Pig	1:2000	I8510 (Sigma)
ITGA1*	Rabbit	1:200*	22146-1-AP (Proteintech)
KLF9*	Mouse	1:150*	CF808444 (Origene)
MAFA	Rabbit	1:200	BLR067G (Bethyl)
NTPDase3	Mouse	1:50	hN3-B3s (http://ectonucleotidases-ab.com/)
SIX2	Rabbit	1:500	11562-1-AP (Proteintech)
SST	Mouse	1:100	sc-74556 (Santa Cruz)
UCN3	Rabbit	1:2000	Code PBL #7218, 01/12/11 bleed (from Dr. Paul Sawchenko and the Salk Institute)
ZNT8	Rabbit	1:2000	ab244550 (Abcam)

Target	Color	Dilution	Product
Anti-Mouse	488	1:800	A11001 (Life Technologies)
Anti-Mouse	568	1:800	A11031 (Life Technologies)
Anti-Mouse	647	1:800	A21235 (Life Technologies)
Anti-Rabbit	488	1:800	A21206 (Life Technologies)
Anti-Rabbit	568	1:800	A11011 (Life Technologies)
Anti-Guinea Pig	488	1:800	A11073 (Life Technologies)

***Required signal amplification (Tyramide SuperBoost™ Kit)**

Alexa Fluor™ 488 Tyramide SuperBoost™ Kit, goat anti-rabbit IgG: ThermoFisher B40922

Alexa Fluor™ 594 Tyramide SuperBoost™ Kit, goat anti-mouse IgG: ThermoFisher B40915

Supplemental Table 6: Primers used for QPCR

Gene	Product Number
ARNTL	Hs00154147_m1
CHGA	Hs00154441_m1
CHGB	Hs01084631_m1
ENTPD3	Hs00154325_m1
ERO1LB	Hs00219877_m1
FAM159B	Hs00971129_m1
G6PC2	Hs01549772_m1
GCK	Hs01564555_m1
GCG	Hs01031536_m1
HDAC9	Hs01081558_m1
HOPX	Hs05028646_s1
IAPP	Hs00169095_m1
INS	Hs02741908_m1
ITGA1	Hs00235006_m1
KLF9	Hs00230918_m1
MAFA	Hs01651425_s1
NR1D1	Hs00253876_m1
OC2	Hs00191477_m1
SIX2	Hs00232731_m1
SIX3	Hs00193667_m1
SYT4	Hs01086433_m1
SLC2A1	Hs00892681_m1
SLC2A2	Hs01096908_m1
SLC30A8	Hs00545183_m1
SST	Hs00356144_m1
SYT4	Hs01086433_m1
UCN3	Hs00846499_s1

Supplemental Table 7: Sources of reagents used for differentiation

Product	Product Number	Supplier
Activin A	338-AC-050	R&D
CHIR-99021	NC9785126	ThermoFisher
Vitamin C (Ascorbic Acid)	A4544	Millipore Sigma
FGF10	PHG0204	ThermoFisher
LDN (LDN183189)	S2618-2MG	ThermoFisher
RA (Retinoic Acid)	R2625	Millipore Sigma
SB-431542	NC9993293	Stemgent
FGF7	251-KG-050	ThermoFisher
SANT-1	S4572-5MG	Cayman Chemical
TPB	565740	Millipore Sigma
ITS-X	51500056	ThermoFisher
Nico (Nicotinamide)	N0636-100G	Millipore Sigma
Heparin	H3149	Millipore Sigma
T3	T6397	Millipore Sigma
Alk5i II	ALX-270-445-M005	Enzo
Compound E (Comp E) (GSI XXI)	82602-302	ThermoFisher
Forskolin	F6886-10MG	Millipore Sigma
Exendin-4 (Ex-4)	E7144-.1MG	Millipore Sigma
R4028	NC0532629	ThermoFisher
BMP4	314-BP-050	R&D
Trolox	648471-500MG	Millipore Sigma
NAC (N-Acetyl Cysteine)	A9165-5G	Millipore Sigma
Bay-K (R(+) Bay K 8644)	04-0013	Stemgent
MK-801	M107-5MG	Millipore Sigma
ZnSO4	Z0251-100G	Millipore Sigma
Glutamax	35050061	ThermoFisher
BSA	A7030-50G	Millipore Sigma
N2	17502001	ThermoFisher
B27	17504044	ThermoFisher
MTG (1-Thioglycerol)	M6145-25ML	Millipore Sigma
P/S (Penicillin/Streptomycin)	15140122	ThermoFisher
MCDB131	10372019	ThermoFisher
CMRL	99-603-CV	Cellgro

Supplemental Methods

Image Quantification

For quantification of SIX2 in images (Supplemental Fig. 5), to clean up non-specific signal, merged images were generated between the DAPI and SIX2 channels to establish the "SIX2+ nuclei" overlap. The merged image clearly revealed localization of SIX2 to the nuclei of the adult islets only and absence in the fetal tissues, in correlation with previous studies using the same antibody (Arda et al. 2016).
